# The role of microbiota in kelp gametophyte development and resilience to thermal stress

**DOI:** 10.1111/jpy.70018

**Published:** 2025-04-29

**Authors:** Reina J. Veenhof, Alexander H. McGrath, Curtis Champion, Symon A. Dworjanyn, Ezequiel M. Marzinelli, Melinda A. Coleman

**Affiliations:** ^1^ Faculty of Science and Engineering, National Marine Science Centre Southern Cross University Coffs Harbour New South Wales Australia; ^2^ School of Life and Environmental Sciences The University of Sydney Sydney New South Wales Australia; ^3^ Sydney Institute of Marine Science Mosman New South Wales Australia; ^4^ Fisheries Research, NSW Department of Primary Industries and Regional Development National Marine Science Centre Coffs Harbour New South Wales Australia

**Keywords:** experimental microbial ecology, gametogenesis, holobiont, life history, microbiome, ocean warming, seaweed, thermal tolerance

## Abstract

Ocean warming is driving profound changes in the ecology of marine habitat formers such as kelps, with negative implications for the biodiversity and ecosystem services they support. Thermal stress can disturb associated microbiota that are essential to the healthy functioning of kelp, but little is known about how this process influences early‐life stages. Because kelps have a biphasic life cycle, thermal stress dynamics of adult sporophyte microbiota may not reflect those of the free‐living haploid gametophyte. We investigated the role of microbial disruption under thermal stress on gametophytes of the kelp *Ecklonia radiata* and compared sporophyte and gametophyte microbiota. The microbiota of gametophytes changed significantly when the microbiome was disrupted and under increased temperature (26°C), in which putative generalist bacterial taxa proliferated and bacterial families associated with nitrogen fixation decreased. Concurrently, the survival of gametophytes decreased to <10%, and surviving gametophytes did not become fertile when the microbiome was disrupted. The length of gametophytes decreased under both microbial disruption and thermal stress. Taken together, this suggests that the associated microbiota of *Ecklonia* gametophytes is important for their survival, fertility, and response to warming. Gametophyte and parental sporophyte microbiota were also distinct from the water column but not each other, suggesting vertical transmission of microbiota from one life stage to the next. This study furthers our understanding of the role of microbiota in gametophyte stress tolerance as well as the acquisition of microbiota, which may prove vital in protecting and increasing the stress resilience of these foundation species.

**Abbreviations**
AIC: Akaike information criteria
ASVs: amplicon sequence variants
dbRDA: distance‐based redundancy analysis
FSW: 1 μm filtered, UV‐sterilized seawater
GLM: generalized linear models
GLMMs: generalized linear mixed effect models
nMDS: non‐metric multi‐dimensional scaling
PC: procedural control
PCR: polymerase chain reaction
PERMANOVA: permutational multivariate analysis of variance

## INTRODUCTION

Kelp forests are globally important ecosystems that support temperate reefs, which in turn provide numerous ecosystem services (Dayton, 1985; Eger et al., 2023). Kelps (seaweeds from the order Laminariales) are primary producers and considered ecosystem engineers, as they provide habitat, nutrition, and shelter to a vast array of species (Fragkopoulou et al., 2022). However, ocean warming is threatening these important habitat‐forming species, resulting in concurrent losses of associated ecosystem benefits (Pessarrodona et al., 2021; Wernberg, Krumhansl, et al., 2019). For example, ocean warming can decrease growth and primary production in kelps, negatively impact their fertility, and alter ecological interactions (Bennett et al., 2024; Franke et al., 2024; Vergés et al., 2019). Effective management strategies to improve thermal resilience in kelps will rely on creative and holistic solutions to counter or mitigate thermal stress (Marzinelli et al., 2024; Wood et al., 2019).

Seaweeds, including kelps, do not exist as solitary organisms but rather as holobionts (Egan et al., 2013), defined as the eukaryotic algal host and its associated microbial community (McFall‐Ngai et al., 2013; van der Loos et al., 2019). There is strong evidence that microbial communities (here broadly defined as microbiota) associated with macroalgae such as kelps are important for their normal function and development (Li et al., 2022; McGrath et al., 2024; Nakanishi et al., 1996; Younker et al., 2024) as well as their responses to environmental change (Dittami et al., 2016; Qiu et al., 2019; Saha et al., 2020; Weigel & Pfister, 2019). There is growing interest in understanding the role microbiota have in the stress tolerance of kelps for both restoration and cultivation, and the potential use of probiotic inoculation to increase stress tolerance (Li et al., 2023; Marzinelli et al., 2024; Saha et al., 2024). However, kelps have a complex, biphasic life cycle, which means that to understand the response of the holobiont to temperature stress, all life history stages should be assessed (Saha et al., 2024).

Kelp juvenile stages consist of two phases: the microscopic gametophyte, which can grow into juvenile sporophytes after fertilization (Schiel & Foster, 2006). Juvenile sporophytes then grow into adult sporophyte kelps. Although there is a considerable amount of literature on the reproductive biology of kelps (Liu et al., 2017; Schiel & Foster, 2006), our knowledge of the composition, function, and acquisition of the microbiota associated with kelp gametophytes is far less (Veenhof et al., 2022). Discrete life history phases can develop distinct associated microbiota (Bonthond et al., 2022; Lemay et al., 2018), and in both red and green seaweeds, bacterial communities play a significant role in the normal development of spores and zygotes (e.g., Provasoli & Pintner, 1980; Singh et al., 2015; Weiss et al., 2017). For example, the sporophytes and gametophytes of red seaweed *Mastocarpus* spp. retain a distinct “core” of microbiota (Lemay et al., 2018). Cultivated juvenile sporophytes in the nursery stage and adult sporophytes deployed at sea also harbor distinct microbial communities in *Alaria marginata* and *Saccharina latissima* (Davis et al., 2023). This shows that the gametophyte microbiota may be distinct from that of the adults, which could potentially fulfill different functional roles compared to adult sporophytes microbiota.

Furthermore, if adult sporophytes transmit their microbiota to gametophytes, then this could be a target life stage of microbial inoculation in the future (Li et al., 2023). However, several knowledge gaps exist that hinder our understanding of how and if this might work for kelps at different life history stages. Firstly, the role of microbiota in kelp gametophyte function and survival is largely unknown. Secondly, the functioning of microbiota under environmental stress is also poorly understood but could be critical, given that increased temperatures can change the host‐associated microbiota in adult kelps, leading to bleaching, disease, and loss of photosynthetic efficiency (Castro et al., 2024; but see Delva et al., 2023; Mancuso et al., 2023; Minich et al., 2018; Qiu et al., 2019). Finally, there is little understanding of the mode of acquisition of gametophyte microbiota. Recent work has shown that vertical transmission of microbiota in macroalgae varies significantly among taxa, ranging from little to no transmission in *Ulva* to a non‐selective or strongly selective transmission in the fucoids *Phyllospora comosa* and *Hormosira banksii*, respectively (Syukur et al., 2024). It has been suggested that the microbiota is passed from sporophyte to gametophytes in the kelp *Macrocystis pyrifera*, but this has not been directly tested by comparing sporophyte and gametophyte microbiota in kelps (Osborne et al., 2023).

Here, we used the gametophytes of the kelp *Ecklonia radiata* (hereafter *Ecklonia*) to study (1) the influence of associated microbiota on temperature responses of gametophytes and (2) the mode of microbiota acquisition. *Ecklonia* is the most abundant forest‐forming kelp on Australia's Great Southern Reef, covering extensive interconnected temperate reefs (Wernberg, Coleman, et al., 2019). As such, it underpins invaluable biodiversity as well as economic and social co‐benefits (Bennett et al., 2016). Increasing ocean temperatures and marine heatwaves threaten *Ecklonia*'s survival, and decreased survival can result in increased patchiness in *Ecklonia* forests, as well as range contractions, local extinctions, and loss of diversity (Coleman et al., 2020; Layton et al., 2020; Wernberg et al., 2013, 2018). The microbiota of healthy *Ecklonia* sporophytes has been well studied, showing stability across 30° of latitude, suggesting active selection and maintenance of the microbiota by the *Ecklonia* host (Marzinelli et al., 2015). Increased temperature can stress the holobiont, resulting in dysbiosis, a shift in microbiota with negative effects for the host (van der Loos et al., 2019), which in turn can result in a diseased, bleached *Ecklonia* host (Castro et al., 2024; Qiu et al., 2019). In juvenile *Ecklonia* sporophytes, thermal stress changes the microbial composition, although a disruption of the microbiota did not change the thermal stress response in the host (Vadillo Gonzalez et al., 2024). However, to date, there is no understanding of the composition, role, or response of microbes on the gametophytes of this key kelp species.

Our first aim was to characterize the microbiota on gametophytes of *Ecklonia radiata* under separate thermal treatments (18, 22, and 26°C, respectively) and to assess performance (survival, length, and reproductive development) of gametophytes under thermal stress with and without a disrupted microbiota by using antimicrobials (McGrath et al., 2024; Vadillo Gonzalez et al., 2024). Our second aim was to compare parent and gametophyte microbiota. We predicted that gametophytes with a disrupted microbiota would perform poorly compared to undisrupted individuals and that this effect would be exacerbated under thermal stress. We also predicted that the microbiota of gametophytes would be more similar to that of the adult kelp than to that of the surrounding seawater and that core microbial taxa would be transmitted from adult to gametophyte.

## MATERIALS AND METHODS

### Culturing algal material

Eight adult *Ecklonia radiata* were taken from two sites (inside and just outside the Coffs Harbour Marina, separated by Muttonbird Island; 30°18′ S 153°08′ E) on September 1, 2023. These sites were chosen to potentially capture different kelp microbiomes, as the sites differed in exposure and anthropogenic influence; although given these factors were not replicated, we treated sites as random. At the same time, water samples (*n* = 3) were taken at each site and filtered through a sterile 2‐μm filter for microbiota analysis. Water filters and kelp were transported back to the laboratory on ice within 1 h. Adult plants were examined for fertile tissue, and four fertile adults per site were selected. Each adult was swabbed in the mid‐thallus with a sterile swab for 1 minute on a 5 × 5 cm^2^ area (see Marzinelli et al., 2015; Qiu et al., 2019), adjacent but not on the fertile area where spores were to be released. The water filters, adult swabs, and swab blank controls (*n* = 3) were immediately frozen at −80°C.

Fertile tissue was excised from the adult plants and left to dry at 18°C. Spore release was induced from four adults per site by submerging fertile tissue in 1 L of 1‐μm filtered, UV‐sterilized seawater (FSW) for 1 h while gently stirring. The spore solution was checked using a hemacytometer counting chamber, and 40 mL of spore solution was allocated to 50‐mL glass beakers containing a 12 × 12 mm coverslip for settlement, resulting in a final spore density of 100 spores · mm^−2^ · replicate^−1^. Beakers were wrapped in parafilm to counteract evaporation and left to settle overnight at 18°C. Spores germinated and grew into small gametophytes within 5 days under a 12:12 daylength cycle and 20 μmol photons · m^−2^  · s^−1^ light intensity, at which point they were allocated randomly to treatment levels as described below.

### Experimental setup

The effect of temperature and microbiota disruption on gametophyte cultures was tested using a full factorial design where three levels of temperature (18, 22, and 26°C) and two timepoints for quantifying growth (1 and 3 weeks) were crossed with three levels of microbiota treatment (disrupted, procedural control, and control). In addition, site (two levels) was included as a random factor. Each combination of temperature, microbiota treatment, site, and timepoint was replicated five times, resulting in a total of 180 experimental independent replicates. Individual replicates were available for each timepoint, allowing independent comparison between timepoints and destructive sampling. However, swabs to characterize microbiota were only performed at Week 3, resulting in a total of 90 samples of gametophyte microbiota for analyses.

Temperature levels (18, 22, and 26°C) were chosen to reflect average winter, summer, and projected climate change temperatures for the source population off Coffs Harbour (Wijffels et al., 2018). Commercially available betadine solution (10% povidone iodine) was chosen to remove the putative gametophyte‐associated microbiota in the disrupted treatment, which was demonstrated to be an effective method in trials and is known to be more effective at lowering bacterial abundance than other antimicrobial (e.g., antibiotics) treatments in *Ecklonia* juveniles (Vadillo Gonzalez et al., 2024). Betadine (povidone iodine) was added at 5% to FSW containing half‐strength F/2 (AlgaBoostTM 2000×, AusAqua Pty Ltd., Wallaroo, SA, Australia). Povidone was used as a procedural control (PC) for any side effects of betadine on gametophyte growth independent of microbiota removal. Povidone is the chemical used to deliver iodine, which is the active substance in betadine solutions. The povidone control solution was made up at 8% povidone in MilliQ water, mimicking the concentration of povidone in betadine. This was added at 5% to FSW containing half‐strength F/2. The control treatment had no added solutions to FSW except for half‐strength F/2. Trials prior to experimental setup showed no negative effect of 1%, 2%, and 5% betadine in culture water on gametophyte survival over a 2‐week period, while the 5% solution effectively altered microbial composition. Each treatment was refreshed with FSW with the appropriate additions after 5 days of growth and placed in their respective temperature treatments (18, 22 and 26°C). Temperatures were maintained in temperature‐controlled chambers with light provided at 20 μmol photons · m^−2^ · s^−1^ on a 12:12 daylength cycle, which is within the optimum light range for growth for *Ecklonia* (Novaczek, 1984).

After 1 week, cultures were destructively sampled by taking the cover slip out of the beaker and taking three photographs at ×200 magnification using a MIchrome 20 Color Microscope camera mounted on a stereo microscope (Olympus BX53). After 3 weeks, cultures were sampled by taking out the coverslip for photography as above. From the photographs taken at Weeks 1 and 3, abundance · mm^−2^ of view relative to starting densities (100 spores · mm^−2^) and length (μm) and sex ratio (numbers of males and females) were calculated, as well as the presence of oogonia and eggs on female gametophytes and occurrence of juvenile sporophytes, if any. The remaining gametophytes in the beakers were swabbed immediately after photographing with sterile cotton buds for 1 min. In addition, culture water samples (*n* = 2) were taken from the three temperature treatments through a 2‐μm filter, as well as air controls (*n* = 3). Swabs and water filters were immediately frozen at −80°C.

### DNA extraction and sequencing

Microbial DNA was extracted from each swab sample in a randomized order to avoid introducing any bias due to order and time of processing, using a Powersoil DNA Isolation kit (Qiagen) following the manufacturer's protocol. DNA extracts were quantified using spectrophotometry (Nanodrop 1000) and stored at −20°C until sequencing. The extracted DNA samples were amplified with polymerase chain reaction (PCR) using the 16S primers 341 (F) (5′‐CCTACGGGNGGCWGCAG‐3′) and 805(R) – (5′‐GACTACHVGGGTATCTAATCC‐′3), containing the V3‐V4 regions of the bacterial and archaeal 16S rRNA gene (Klindworth et al., 2013). Both positive (with known DNA sequences) and negative controls (nuclease‐free water, control swabs) were used. The negative controls did not amplify DNA, suggesting no contamination of swabs or during extraction and amplification. Agarose gel electrophoresis and Nanodrop 1000 were used to ensure the quantity and quality of the amplicons before they were sent for sequencing via the Illumina MiSeq 2000 platform at the Ramaciotti Centre for Genomics (University of New South Wales, Sydney).

### Sequencing and bioinformatics

Raw sequences were received from the sequencing center as demultiplexed paired‐ended sequences per sample. UNOISE was used to remove chimeras and produce amplicon sequence variants (ASVs), that is, amplicon sequence variants at a unique (0% distance) sequence level (Edgar, 2017). DADA2 was used to map the original reads back to ASVs, generating a table of 11,293 ASVs (Callahan et al., 2017). The ASV sequences were searched with BlastN against the SILVA SSU Ref NR99 database for taxonomic classification and to remove chloroplasts; the Genome Taxonomy Database (GTDB) was then used for taxonomic assignment. Singletons and low abundance taxa (<0.01% of reads) were removed from the dataset prior to statistical analyses, resulting in 9569 ASVs.

Total abundance of the 16S rRNA gene was quantified for each sample by qPCR using the primers 341F/805R (Thijs et al., 2017). Gene amplification and analysis were performed using the QuantStudio 3 thermocycler (Thermo Fisher with PrimeTime® Gene Expression Master Mix, Integrated DNA Technologies) and associated software. The reaction conditions for amplification of DNA were 50°C for 2 min, 95°C for 10 min, and 40 cycles of 95°C for 15 s and 60°C for 1 min. The final gene copy number per sample was corrected for the total extraction volume, the surface area, the dilution factor, and DNA yield per sample (see Nappi et al., 2023) and was used to estimate absolute abundances of ASVs.

### Data analysis: Microbial community

All statistical analyses were carried out in R (R Core Team, 2022). To account for uneven sequencing depth among samples, data were normalized using total 16S rRNA gene reads per sample of the V3‐V4 region, which were calculated using qPCR (Nappi et al., 2023). Alpha diversity measures of richness (i.e., the number of unique sequences) and Simpson's diversity index were calculated using the vegan package (Oksanen et al., 2022), and differences among treatment (three levels, fixed), temperature (three levels, fixed, crossed), their interaction, and site (two levels, random) were examined using a linear model in the GAD package (Silva, 2014).

To determine differences in the structure of the associated bacterial assemblages, the normalized ASV data were analyzed using permutational multivariate analysis of variance (PERMANOVA, Anderson & Walsh, 2013) using the vegan package (Oksanen et al., 2022), with the factors treatment, temperature, their interaction, and site as above. Analyses were based on Bray–Curtis dissimilarities between sample pairs calculated on square‐root transformed absolute (qPCR normalized) abundances of ASVs. For significant interactions, or for main effects where interactions were not significant, pairwise comparisons were performed using the pairwiseAdonis package (Martinez Arbizu, 2022). These data were visualized through non‐metric multi‐dimensional scaling (nMDS) ordinations based on the Bray–Curtis measure on square‐root transformed data.

A distance‐based redundancy analysis (dbRDA; vegan, Oksanen et al., 2022) was used to determine the relationship between host response variables (survival, fertility, sex ratio, length, and abundance) and the structure of the microbiota. A stepwise model selection was undertaken using Akaike information criteria (AIC) to determine the best model fit using host responses only. The abundance of gametophytes was observed to have high multicollinearity (olsrr; Hebbali, 2024) and was removed from the model, with the rest of the factors being log transformed to reduce their skewness. Furthermore, as no data were able to be gathered on the sex ratio of gametophytes within the disrupted treatment at 26°C, this factor was removed from the model. Estimation of linear trends (envfit; vegan; Oksanen et al., 2022) were used to determine the importance of each predictor to overall microbiota structure.

To determine which bacterial taxa's abundance differed among treatments and temperatures, we used multivariate generalized linear models (GLMs) using mvabund (Wang et al., 2012), assuming a negative‐binomial distribution to account for over‐dispersion.

### Data analysis: Gametophyte data

The survival and length of gametophytes was analyzed with generalized linear mixed effect models (GLMMs) using the lme4 package (Bates et al., 2015) with fixed effects of treatment (disrupted, PC, control), temperature (18, 22, and 26°C), and time (Weeks 1 and 3), and site was included as a random factor. The GLMMs were fit with a logistic link function to model the survival (i.e., Poisson distributed response) and sex (i.e., binomially distributed response variable) and an identity link function for gametophyte length (i.e., Gaussian‐distributed response variable). Sex ratio as a response variable was structured in a matrix containing male and female counts for each replicate. Survival was modeled as the number (i.e., counts) of gametophytes, offset by the initial density. Analysis of deviance assessments (Type III Wald chi‐square tests) were applied to the fitted models using likelihood ratios in the package car (Fox & Weisberg, 2019). Post hoc analysis of significant effects was carried out using estimated marginal means with a Tukey correction for multiple testing in the emmeans package (Lenth, 2024). Residual plots were assessed visually to confirm the GLMMs satisfied assumptions of homogeneity of variance.

## RESULTS

### Effect of disruption on gametophyte microbiota

**FIGURE 1:**
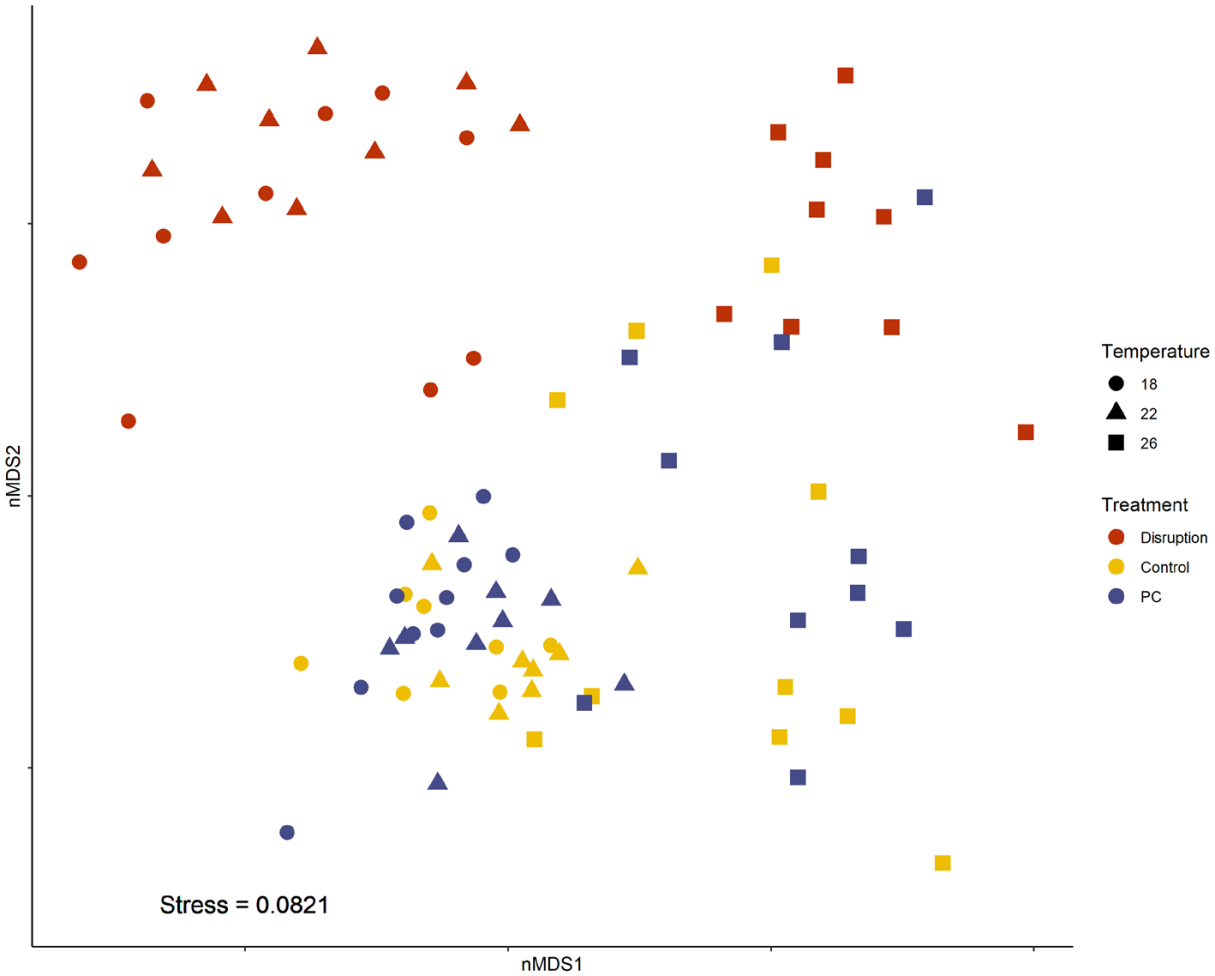
nMDS ordination of *Ecklonia radiata* gametophyte microbiota community structure (Bray–Curtis on qPCR normalized, sqrt‐transformed ASV abundances) across microbiota treatments (disruption with betadine, procedural control with povidone, and control), and temperatures (18, 22, and 26°C) at Week 3 (*n* = 10). Disruption (red), control (yellow), procedural control (navy blue). Stress = 0.08.

The microbiota of gametophytes significantly differed across treatments and temperatures at Week 3 (Figure 1, Table S1, pseudo‐*F*_4,74_ = 0.68, *p* < 0.04). Microbial disruption caused significant changes in the microbial community structure compared with PC and controls (which did not differ from each other) in all temperatures except 26°C (Figure 1; Table S1). The microbial community structure associated with gametophytes within the PC treatment did not significantly differ from controls in all temperatures and across all treatments. However, all treatments (disruption, PC, and controls) within the 26°C temperature treatment differed from their equivalent treatments in 18 and 22°C (Figure 1; Table S1). Distance‐based redundancy analysis (dbRDA) showed that survival of gametophytes was significantly related to bacterial community structure (Figure S1, Table S2, survival, *F*_1,72_ = 1.641, *p* < 0.01). The fertility and length were not significantly related to microbiota structure (Table S2).

**FIGURE 2:**
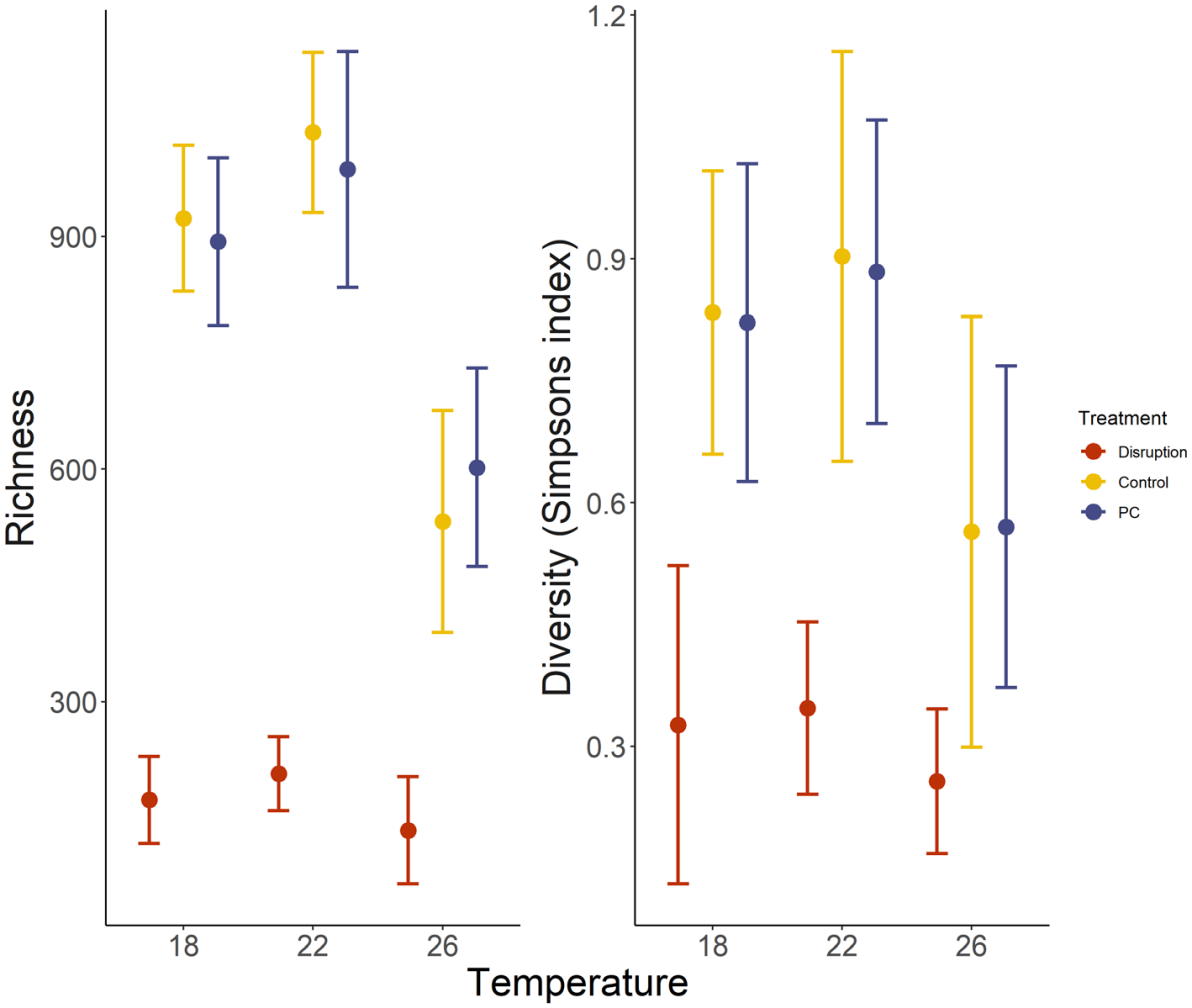
Observed richness (left panel) and diversity (Simpsons diversity index—right panel) among replicates within microbial treatment (disruption with betadine, procedural control (PC) with povidone, and control) and temperatures (18, 22, and 26°C). Dots represent the mean and bars represent standard error (*n* = 5).

The disruption of the gametophyte microbiota with Betadine caused significant decreases in ASV richness compared to controls (control and PC) across temperatures (Figure 2, Table S3, *F*_4,86_ = 1.755, *p* = 0.002). Temperatures of 26°C caused a significant decrease in ASV richness across all microbial treatments (Figure 2, Table S3). There were no significant decreases in ASV richness between controls and PC across temperatures (Figure 2, Table S3). Microbial disruption also caused a significant decrease in ASV Simpson's diversity across temperatures and treatments (Figure 2, Table S3, *F*_4,86_ = 1.235, *p* = 0.002). Simpson's diversity was significantly lower for the disrupted treatments, except 26°C, for which there were no significant differences (Figure 2, Table S3).

Generalized linear models identified 204 ASVs associated with the gametophytes (approx. 2.1% of a total of 9569 ASVs, Table S4), and abundances differed significantly among treatments and temperatures. Of those, we observed a significant negative effect of treatment on 11 ASVs in the families Chroococcidiopsaceae, Arenicellaceae, and Colwelliaceae, with abundances of these taxa being lower (31%–58%; Table S4) in the disruption treatments and all within temperatures of 26°C (>32%). Furthermore, there was a significant positive effect of treatment on 16 ASVs belonging to the families Vibrionaceae, Alteromonadaceae, Pseudoalteromonadaceae, and Colwelliaceae (Figure S2; Table S4).

**FIGURE 3:**
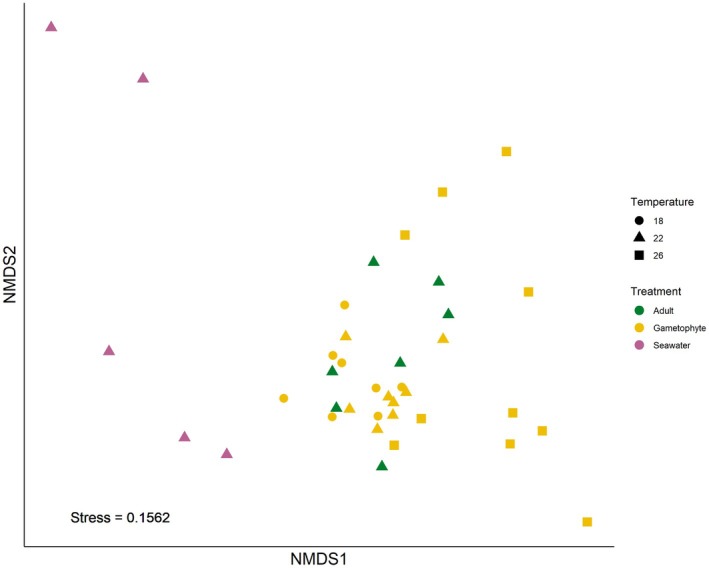
nMDS ordination of *Ecklonia radiata* adult and gametophyte microbiota community structure (Bray–Curtis on qPCR normalized, sqrt‐transformed ASV abundances) compared with seawater. Each dot represents one microbiome replicate of an adult (green, *n* = 7), seawater (purple, *n* = 5), or gametophyte (yellow, *n* = 30). Stress = 0.1562. The gametophyte data for this plot were taken only from the undisturbed (control) treatment.

The structure of the microbiota associated with undisturbed gametophytes and adults differed significantly from the surrounding seawater (Figure 3, Figure S3, Table S5, pseudo‐*F*_2,14_ = 1.64, *p* < 0.001). Observed richness and diversity, however, did not differ (Figure S4). The GLM analyses indicated that adults shared a common group of 139 taxa (>0.01% abundance) with the gametophytes. Twelve members of the class *Cyanophyceae* were identified as being shared between adults and gametophytes (Figure S5). Of these 12, two genera are known symbionts in other marine species, *Acaryochloris* sp. and *Chroococcidiopsis* sp. Many of the remaining shared taxa belonged to the orders Pseudomonadales and Rhizobiales.

### Gametophyte performance

**FIGURE 4:**
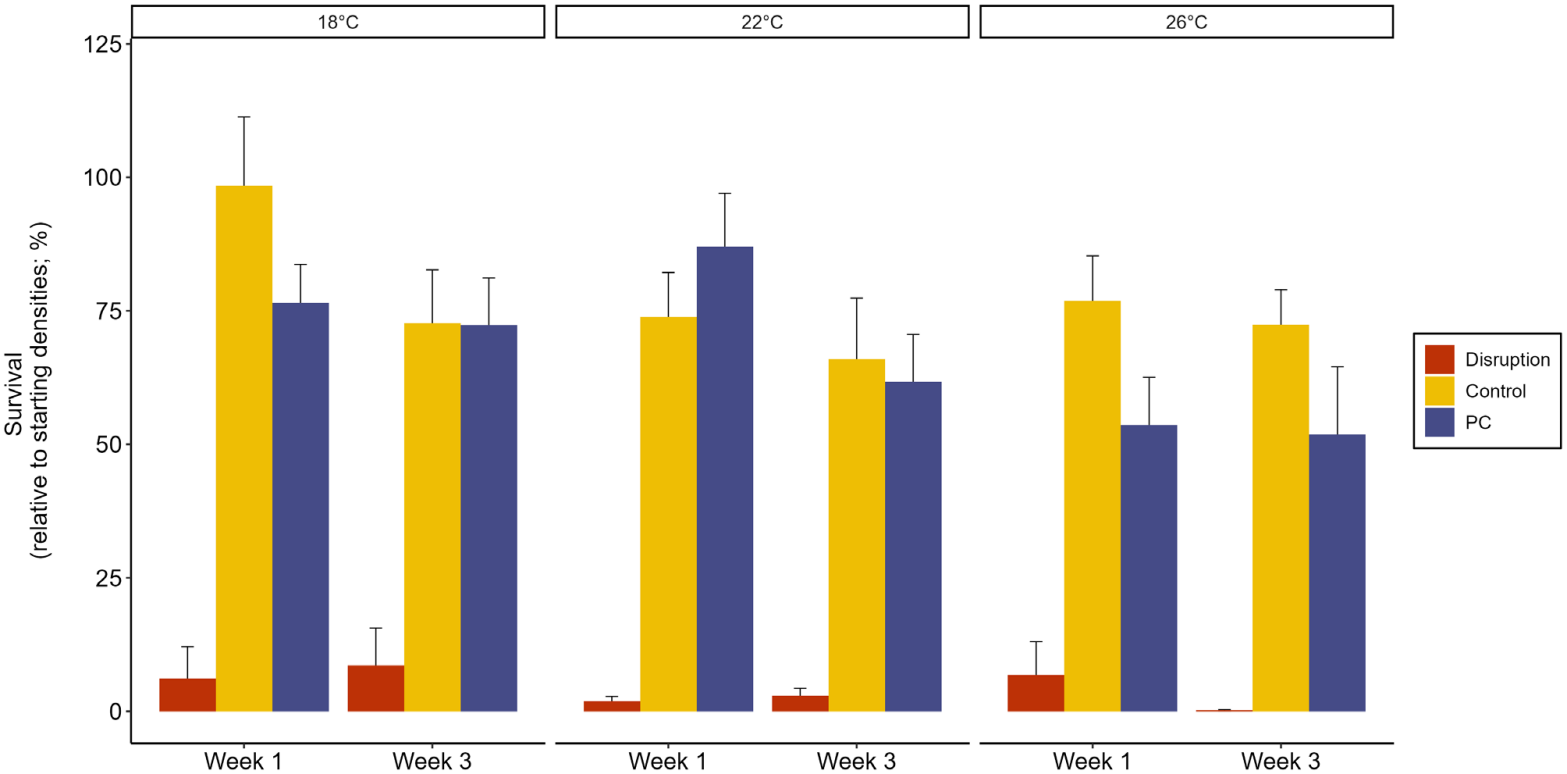
Mean survival rates (calculated as % change in abundance from initial settled spores to gametophytes) of *Ecklonia radiata* gametophytes under separate microbiota treatments (disruption with betadine, procedural control (PC) with povidone, and control), temperatures (18, 22, and 26°C), and timestamp (Week 1 and Week 3). Error bars represent standard error (*n* = 10).

There was a significant interaction among microbiota treatment, temperature, and time of measurement for gametophyte survival (Figure 4, Table 1, χ^2^ = 51.85_4,162_, *p* < 0.001). Post hoc tests showed that at Week 3, survival was significantly lower at 26°C compared to 18°C in the disrupted treatment (from ~10% to 0%; Figure 4, Figure S6, Table 1, *p* < 0.001), but there was no significant difference in survival across temperature treatments for the control treatment (Figure 4, Figure S6, Table 1). Post hoc analysis showed that at Week 3, the PC also had lower survival at 26°C compared to 18°C (from ~70% to 50%; Figure 4, Figure S6, Table 1, *p* = 0.010). Gametophytes in the disruption treatment had an overall significantly lower survival (4%) compared to in the PC (67%) and control (76%) treatments across all factor combinations (Figure 4, Figure S6, Table 1, *p* < 0.001, post hoc). Survival did not significantly differ between the control and PC treatments, except for lower survival in PC by ~20% at 18°C in Week 1, and at 26°C across both weeks (Figure 4, Figure S6, Table 1, *p* < 0.001, post hoc).

**TABLE 1:** Type III Wald chi‐square test of GLMs modeling survival, length, and sex ratio of *Ecklonia radiata* gametophytes under different microbiota treatments (disruption with betadine, procedural control with povidone and control), temperature (18, 22 and 26°C), and timepoint (Week 1 and Week 3) including “site” as a random factor. Significant results at the *α* = 0.05 level are indicated in bold.

Variable	Factor	*χ* ^2^	df	*p*
Survival	Treatment (Tr)	447.726	2	**<0.001**
	Temperature (Te)	25.181	2	**<0.001**
	Timepoint (Ti)	4.211	1	**0.040**
	Tr × Te	89.784	4	**<0.001**
	Tr × Ti	21.533	2	**<0.001**
	Te × Ti	28.427	2	**<0.001**
	Tr × Te × Ti	51.854	4	**<0.001**
All levels of Time and Temperature: Control = PC > Disruption Week 1, disruption: 18°C = 26°C > 22°C | Week 3, disruption: 18°C > 22°C > 26°C Week 1, control: 18°C > 22°C = 26°C | Week 3, control: 18°C = 22°C = 26°C Week 1, PC: 18°C = 22°C > 26°C | Week 3, PC: 18°C = 22°C > 26°C				
Length	Treatment (Tr)	7.388	2	**0.025**
	Temperature (Te)	0.463	2	0.793
	Timepoint (Ti)	13.843	1	**<0.001**
	Tr × Te	0.812	4	0.937
	Tr × Ti	0.423	2	0.809
	Te × Ti	11.606	2	**0.003**
	Tr × Te × Ti	8.195	4	0.085
Treatment: Control > PC > Disruption Week 1: 18°C = 22°C = 26°C Week 3: 18°C = 22°C > 26°C				
Sex ratio	Treatment (Tr)	1.258	2	0.533
	Temperature (Te)	0.632	2	0.729
	Timepoint (Ti)	0.048	1	0.827
	Tr × Te	2.078	4	0.722

**FIGURE 5:**
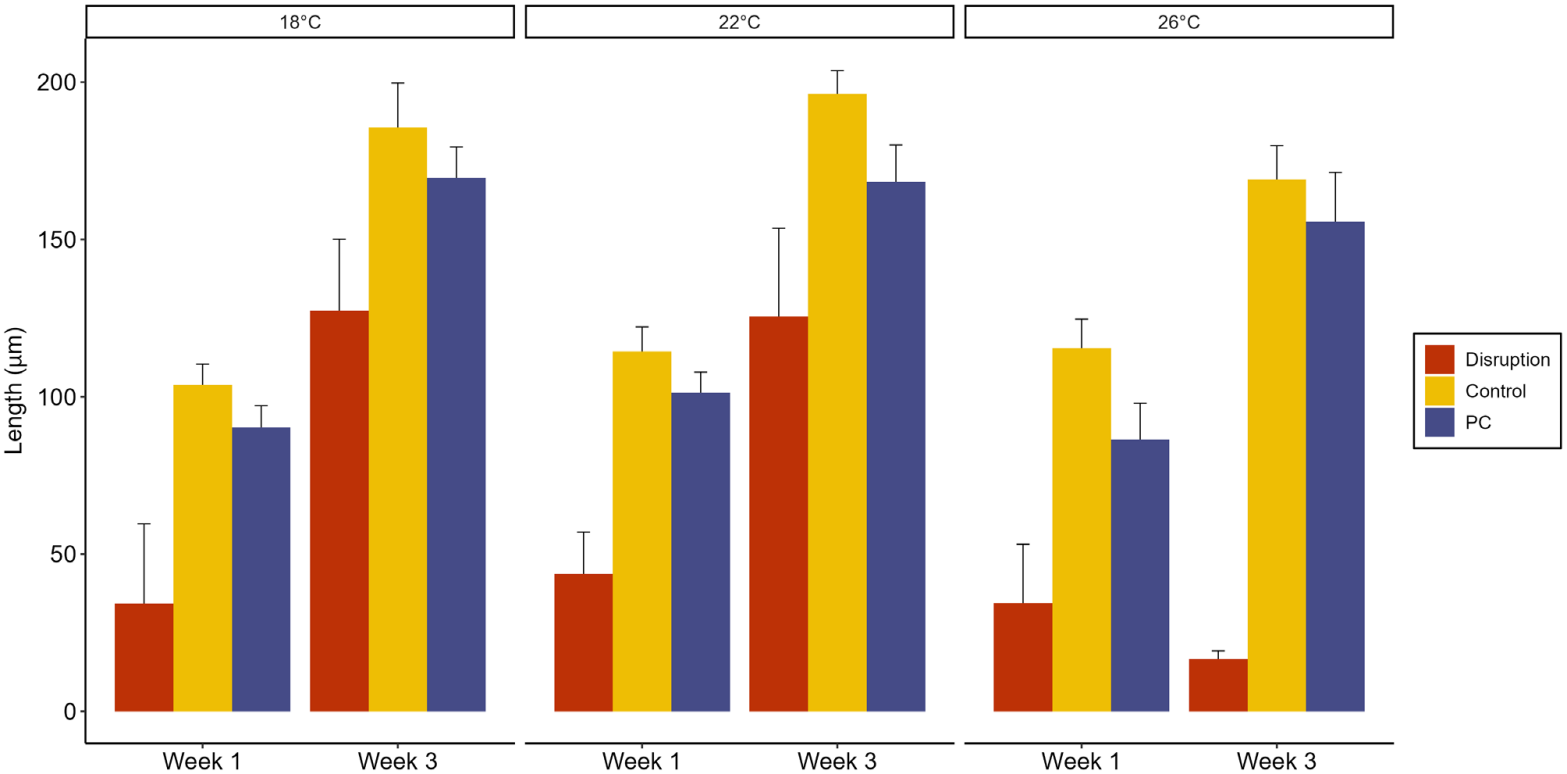
Mean length (μm) of *Ecklonia radiata* gametophytes under separate microbiota treatments (disruption with betadine, procedural control (PC) with povidone, and control), temperatures (18, 22, and 26°C) and timepoints (Week 1 and Week 3). Error bars represent standard error (*n* = 10).

Gametophyte length (averaged over time and temperature) was lowest in the microbial disruption treatment (81 μm) followed by the PC (128 μm), and controls had the greatest gametophyte length (147 μm; Figure 5, Table 1, χ^2^ = 7.39_2,162_, *p* = 0.02). A significant interaction between temperature and time (Figure 5, Table 1, χ^2^ = 11.16_2,162_, *p* = 0.003) showed that length, averaged over microbial treatment, increased significantly from Week 1 to Week 3 across all temperature treatments (56% increase at 18 and 22°C and a 60% increase at 26°C) and that gametophyte length did not significantly differ between temperature treatments at Week 1, but gametophyte length at 26°C was significantly lower (~10%) than at 18 and 22°C at Week 3 (Table 1, *p* < 0.001, post hoc). Although the three‐way interaction was not significant (Table 1, χ^2^ = 8.20_4,162_, *p* = 0.85), this result seemed mainly driven by a sharp decrease in length in the 26°C disrupted treatment compared to all other treatments at Week 3 (Figure 5).

Sex ratios of gametophytes did not differ significantly between microbial or temperature treatment (Table 1, Figure S7). Because many gametophytes had died by Week 3 and those surviving were hard to identify as either male or female, a lack of data in the specific factor combination of the disruption treatment after 3 weeks at 26°C (Figure S7) caused a singular model fit. Therefore, “Timepoint” was included only as a main effect, which resolved the issue. A mean sex ratio of 0.49 indicates an equal proportion of males and females across treatments (Figure S7).

**FIGURE 6:**
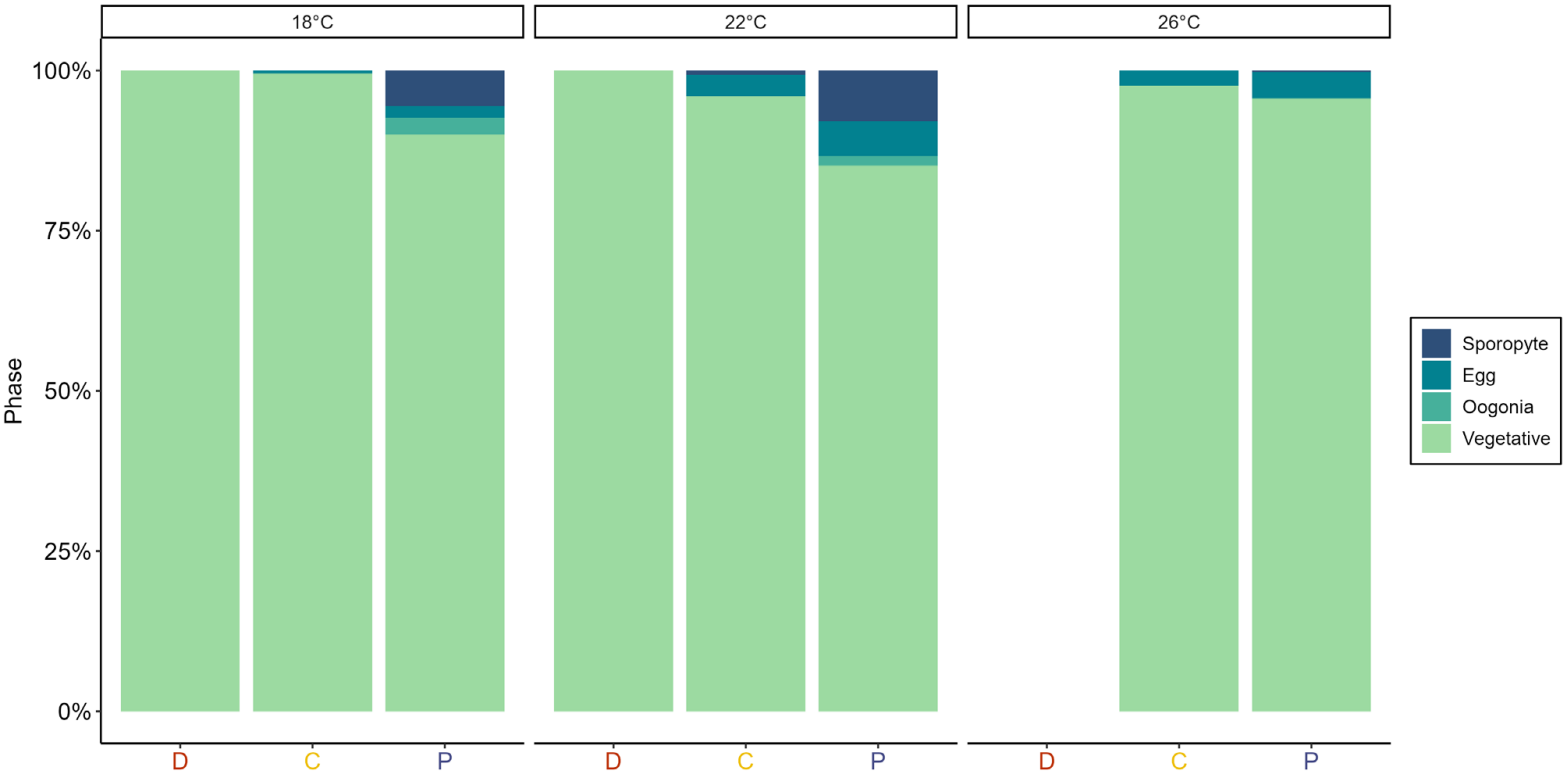
Phase of *Ecklonia radiata* female gametophyte development after 3 weeks as a percentage of total individuals for each treatment group (D = disruption with betadine, P = procedural control with povidone, and C = control) and temperature treatment (18, 22, and 26°C). Gametophytes were classed as either vegetative (no reproductive structures but recognizable female gametophyte), having visible oogonia, having eggs protruding from oogonia, or having juvenile sporophytes developing. Note that no data is visible in the disruption treatment at 26°C, as no recognizable female gametophytes were identified.

Female gametophytes developed from their vegetative form to produce oogonia and eggs after 3 weeks in both control treatments, but not in the disruption treatment (Figure 6). Notably, no recognizably female gametophytes developed at all in the 26°C disruption treatment (Figure 6). Gametophytes in the disruption treatment stayed vegetative across all temperatures. In the control treatments, oogonia appeared after 3 weeks at all temperatures, although in higher numbers at 18 and 22°C and in the PC treatment. The majority of eggs were observed at 18 and 22°C in the PC treatment. Similarly, juvenile sporophytes only appeared in the PC treatment at 18 and 22°C.

## DISCUSSION

The negative effects of ocean warming on habitat‐forming seaweeds, such as kelps, can alter the structure and function of entire ecosystems (Harley et al., 2012; Ji & Gao, 2021; Wernberg et al., 2023). Although the microbiota of seaweeds is critical for their normal function and survival in a changing ocean (see McGrath et al., 2024; Saha et al., 2024; Singh & Reddy, 2014), we know little about the role microorganisms play in the response of kelp early‐life stages to warming. Here, we have shown that disruption to the microbiota of *Ecklonia radiata* gametophytes negatively affected their survival, growth, and fertility, and that this effect was exacerbated by increased temperatures for fertility and survival. This effect likely has implications for the long‐term persistence of these habitat‐forming kelps.

### Gametophyte survival and growth under thermal stress and disruption

The disruption in the microbiota in *Ecklonia* gametophytes caused a significant decrease in host survival across all temperature treatments, which was exacerbated at higher temperatures, nearing 0% survival. Although some decreased survival was also noted in the procedural controls at 26°C (from ~75% to 50%), survival rates of both controls were comparable to previous studies, where warm‐edge *Ecklonia* gametophytes have had high (50%–75%) survival up to 27°C (Veenhof et al., 2024). Additionally, length was lower in the disrupted treatments compared to the controls. The strong negative effect of a disrupted microbial community on survival and length points to a significant role for microbiota in normal gametophyte development.

The disruption treatment caused significant decreases in 27 ASVs, primarily from the classes Cyanophyceae and Gammaproteobacteria. The former of these classes (Cyanophyceae) is known microbial consorts in sponges (Hinde et al., 1994; Usher, 2008), corals (Bourne et al., 2016; Carpenter & Foster, 2002), and macroalgae (Penhale & Capone, 1981). They are often involved with the nitrogen‐acquisition capabilities of their hosts (Fiore et al., 2010; Mohr et al., 2021). The potential loss of this pathway for nitrogen acquisition through the removal of ASVs may reduce nitrogen availability for *Ecklonia* gametophytes, resulting in reduced growth and survival. Indeed, the main bacterial ASVs associated with increased survival in the gametophytes belonged to the families Phyllobacteriaceae and Acaryochloridaceae. The latter of these two families is a symbiont within several marine benthic organisms (Behrendt et al., 2018; Ohkubo & Miyashita, 2012) and contains chlorophyll *d* as its primary pigment, which allows it to use far‐red light for photosynthesis (Komárek, 2016). If a symbiont of *Ecklonia* gametophytes, then it could enable light capture and nutrient cycling within shaded conditions under adult canopy, increasing shade adaptation in early‐life stages (Altamirano et al., 2003).

Changes in the disrupted treatment also characterized increases in 177 ASVs, primarily from the families Vibrionaceae, Alteromonadaceae, Flavobacteriaceae, and Rhodobacteriaceae (Figure S2), which are often classed as “weedy,” opportunistic, and transient taxa, and several species have been related to pathogenicity (Case et al., 2011; Ling et al., 2022; Liu et al., 2019; Vadillo Gonzalez et al., 2024; Vijayan et al., 2019; Zozaya‐Valdés et al., 2017). The virulence of bacteria is commonly regulated by temperature through several diverse mechanisms involving DNA, RNA, or thermoregulatory proteins (Klinkert & Narberhaus, 2009). As sea‐surface temperature increases and host defenses decrease as a result (Franklin & Forster, 1997; van de Poll et al., 2002), gametophytes may become more susceptible to temperature‐regulated pathogens, leading to decreased growth and survival.

Distance‐based redundancy analyses further showed a strong significant effect of host survival on the microbiota structure across temperatures. However, as changes in survival in the host were strongly related to microbial treatment, this observation may have been confounded by the treatment effect. Contrastingly, in *Ecklonia* juvenile sporophytes there was no effect of disrupted microbiota on the host growth rate or morphology, and juvenile performance was equally low under heat stress (Vadillo Gonzalez et al., 2024). In *Ecklonia* adult sporophytes, temperature‐induced change in microbiota also did not change *Ecklonia* morphology, although acidification did (Qiu et al., 2019). This difference in host response can point to a stage‐specific influence of the microbiota on host functioning, in which the microbiota may play a larger role in thermal tolerance in gametophytes than in subsequent life stages. This may, in part, explain the higher thermal tolerance of *Ecklonia* gametophytes compared to adults and juvenile sporophytes (Mohring et al., 2014; Schwoerbel et al., 2024; Veenhof et al., 2024), although further research is necessary to untangle the causal effects of microbiota and temperature stress on host performance in *Ecklonia* (Marzinelli et al., 2024). One example would be growing gametophytes under fully axenic conditions and re‐inoculating them with some of the 27 ASV strains that decrease under thermal stress to assess their function, although culturing strains observed on macroalga is currently still challenging.

### Impact of microbial treatment on fertility

The strong negative effect of microbial treatment on gametophyte fertility points to the potential role of gametophyte‐associated microbiota in kelp recruitment. No female gametophyte produced oogonia or eggs in the disrupted microbial treatment, and this effect was exacerbated at higher temperatures, suggesting that ocean warming may inhibit gametogenesis in kelps, resulting in recruitment failure. Interestingly, the highest number of juvenile sporophytes occurred in the procedural control containing povidone. Although povidone contains nitrogen, it is unlikely that any nutritional benefit was derived directly from povidone, as the molecule is highly stable (Barabas & Brittain, 1998; Schenck et al., 1979). However, microbial activity could have liberated some available nitrogen from the molecule, which can stimulate fertility in kelp gametophytes (Carney & Edwards, 2010; Chen et al., 2023; Martins et al., 2017). Alternatively, the slightly lower density of gametophytes in the PC treatment could have caused increased fertility, as lower gametophyte abundance increases fertility in *Ecklonia* (Schwoerbel et al., 2022).

To date, there is limited research on the role of microbiota on kelp fertility (Davis et al., 2023; Morris et al., 2016; Osborne et al., 2024), but its importance in recruitment of other seaweeds has been recognized, as well as its role in the settlement of many marine larvae (Freckelton et al., 2022). For example, in the brown algal model *Ectocarpus*, no sporophyte development was achieved in axenic cultures, while re‐inoculation with certain strains of bacteria (*Marinobacter adhaerens*, *Roseabacter* sp., *Halomonas* sp., *Antarctobacter* sp., *Methylophaga* sp.) induced reproduction (Tapia et al., 2016). Extensive studies have also shown that *Ulva* sp. develops abnormally in axenic cultures, and certain bacterial strains (e.g., *Roseovarius* sp. MS2 and *Maribacter* sp. MS) can restore regular morphological development (Nakanishi et al., 1996; Provasoli & Pintner, 1980; Weiss et al., 2017). Spore settlement in seaweeds (*Ulva* spp.) is also supported by bacterial communities (Singh & Reddy, 2014; Tait et al., 2005), and the microbial community plays a role in spore release in red seaweeds *Graciliaria* and *Acrochaetium* (Singh et al., 2015; Weinberger et al., 2007). It is therefore plausible that the microbial community on kelp gametophytes plays a role in the successful development of the sexual life phase. If this was the case, a non‐random allocation of microbiota from adults to gametophytes would be expected if there was an evolutionary advantage.

### Acquisition of microbiota in gametophytes

The acquisition of a microbial community by kelps is largely thought to be non‐random and influenced by the pool of microbial communities in the water column from which microbes can colonize kelps. This is described as the “competitive lottery” model sensu Burke et al. (2011), in which both active selection by the host and neutral processes governing the available microbes in the water column result in a “core” microbial community with similar functionality across the host species (e.g., King et al., 2023; Marzinelli et al., 2015; Weigel & Pfister, 2019; Wood et al., 2022). Here, we have shown that the microbial community composition on *Ecklonia* gametophytes is distinct from that of the water column but similar to that of the adult sporophyte. This may either demonstrate retention of the microbial community from the sporophyte to the gametophyte phase, which would be indicative of vertical transmission of the microbial community, or active selection of both sporophyte and gametophytes from the same species for similar microbial communities. This active selection can be due to similar surface exudates or metabolic processes within the kelp species (Saha & Weinberger, 2019). Nursery stages of the cultivated kelps (juvenile sporophytes and gametophytes) *Saccharina latissima*, *Alaria marginata*, and *Macrocystis pyrifera* harbored distinct microbial communities among species and populations (Davis et al., 2023; Osborne et al., 2023) despite being cultured in the same water, pointing to a potential influence of parental microbiota. In contrast, cultivated kelp sporophytes have distinct microbial communities with altered functionality compared with the nursery stages in *S. japonica*, *A. marginata*, and *S. latissima* (Davis et al., 2023; Han et al., 2021).

Kelps may thus transfer their acquired microbial community from adult sporophytes to gametophytes, but the juvenile sporophyte might actively select for microbial symbionts based on differing functional or metabolic needs. Although further research would be needed to test this hypothesis, if supported, this may have some important implications for both restoration and cultivation of kelp and, thus, warrants further investigation. If the gametophyte microbial community is retained during the early stages, then this provides space for inoculating gametophytes with beneficial communities which will give it a “boost” during the early grow‐out phase. The early deployment stage is critical for success in both traditional long‐line seeding methods in kelp aquaculture (Kerrison et al., 2018) and restoration techniques in which seeded substrates are placed on the benthos (Fredriksen et al., 2020). Once established, the sporophytes may select for their microbial community according to environmental and metabolic needs. This is akin to seed biopriming in agriculture, when seeds are inoculated with beneficial microbes in the early‐life stage for increased germination and survival (Mahmood et al., 2016). Although the adult plant subsequently recruits its own root microbiome from the soil, the early‐life stage “boost” can significantly increase yields as well as abiotic stress tolerance (Mahmood et al., 2016; Paparella et al., 2015). This may work similarly in kelps if gametophytes have strong retention of their microbes, supporting the idea that inoculations would be beneficial in the early‐life stage (Li et al., 2023). Research into gametophyte microbial inoculation and in situ testing could confirm these hypotheses.

The adult sporophytes that provided fertile material for the gametophyte cultures used in this study were sourced at two different sites: one putatively “pristine” and one “dirty” site based on their location inside and outside a marina. As these were not replicated, “site” was included in the analysis as a random factor to account for any site‐specific variation. However, it is interesting to point out that both host and microbial responses showed differences among sites. For example, the overall survival rate was lower but fertility higher in the “pristine” sites (Figure S8), and cyanobacteria abundances differed among sites as well (Figure S9). Nearshore versus offshore microbial communities can influence *Macrocytis pyrifera* gametophyte survival: Microbial communities collected in a more urbanized environment had a detrimental effect (Morris et al., 2016). In adult sporophyte communities, urbanization can alter microbial communities (Weigel & Pfister, 2019) and may result in bleaching and disease (Marzinelli et al., 2018). There may, thus, be an effect of urbanization on associated microbial communities, which in turn may alter host performance in kelp gametophytes. However, more research is needed to support this conclusion, particularly using replicated sites sampled across a gradient of urbanization.

Although trials showed no negative effects of Betadine solution on gametophyte survival, the long‐term negative effect iodine could have on both survival and fertility should be considered. Iodine affects microbes through the disruption of normal cell functioning and the inhibition of DNA synthesis, a process which can affect microalgae as well (Ajayan et al., 2023). It is possible that a similar effect would have prevented the growth and sexual development of gametophytes in the disrupted treatments. This could have confounded some of the strong negative effects caused by the microbial disruption treatment. However, within this work we believe the effect is microbially mediated as (1) iodine is readily accumulated in Laminarian tissue and used as an antioxidant (Carrano et al., 2021), and (2) low concentrations (2%) of betadine had no negative effect on juvenile *Ecklonia* cultures within previous work and in trials previous to this work (Vadillo Gonzalez et al., 2024). Therefore, we think that the observed strong, negative effect of iodine on gametophyte development and fertility is due to the disruption of gametophyte‐associated microbiota.

## CONCLUSIONS

Here we have shown that the microbiota associated with *Ecklonia* gametophytes is similar to that of the adult sporophytes and contains some of the “core” microbial taxa present on other marine habitat formers related to N‐fixation. The disruption of this microbiota leads to decreased survival, decreased length, and a complete loss of fertility, which were exacerbated under thermal stress. This shows that gametophyte‐associated microbiota is fundamentally important for the normal function, survival, and growth of *Ecklonia*. Furthermore, gametophyte microbiota may improve performance under thermal stress and should be considered as a potential mechanism for improving resilience to stressors in a warming ocean. As gametophytes here were shown to retain their microbial communities, this may provide a potential pathway for inoculation with beneficial microbes. This can improve chances of survival during the critical early‐deployment stage, leading to increased climate resilience and chances of success in both restoration and aquaculture.

## AUTHOR CONTRIBUTIONS

**Reina J. Veenhof:** Conceptualization (lead); formal analysis (lead); funding acquisition (equal); investigation (lead); methodology (lead); writing – original draft (lead); writing – review and editing (lead). **Alexander H. McGrath:** Conceptualization (lead); formal analysis (lead); funding acquisition (equal); investigation (lead); methodology (lead); writing – original draft (lead); writing – review and editing (lead). **Curtis Champion:** Conceptualization (equal); writing – review and editing (equal). **Symon A. Dworjanyn:** Conceptualization (equal); funding acquisition (lead); writing – review and editing (equal). **Ezequiel M. Marzinelli:** Conceptualization (equal); funding acquisition (lead); methodology (equal); writing – review and editing (equal). **Melinda A. Coleman:** Conceptualization (equal); funding acquisition (lead); methodology (equal); writing – review and editing (equal).

## FUNDING INFORMATION

R.J.V. and A.H.M. are supported by the Holsworth Wildlife Research Endowment. This work contributes to the NSW Primary Industries Climate Change Research Strategy, funded by the NSW Climate Change Fund. Funding was also provided by Australian Research Council grant DP200100201 awarded to M.A.C. A.H.M. was further supported by a scholarship granted by The University of Sydney through the William George Murrell bequest.

## Supporting information


Data S1
