# Supplementary material for: The role of microbiota in kelp gametophyte development and resilience to thermal stress
Source: J Phycol. 2025 Apr 29;61(3):633–49. doi: 10.1111/jpy.70018 (PMC12168109; doi:10.1111/jpy.70018)
Supplement: Supplementary file 1 — Data S1 [file JPY-61-633-s001.docx]

**Supplementary figures**


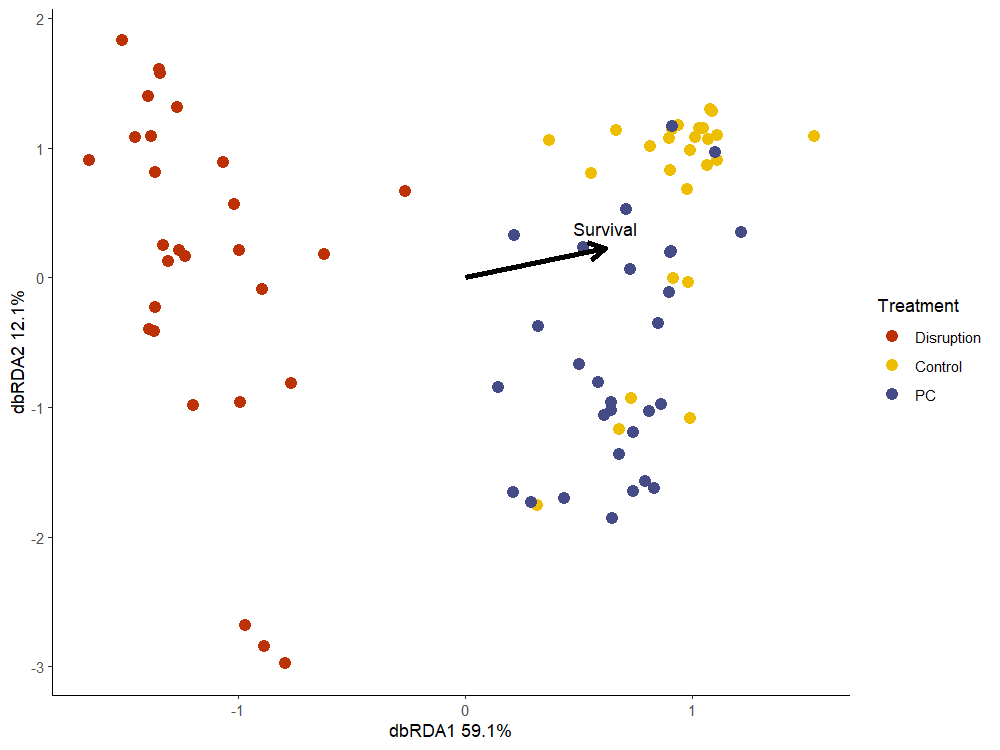


*Figure S1.* dbRDA plot displaying the ordination of microbial community samples (Bray-Curtis on qPCR normalised, sqrt-transformed ASV abundances) based on treatment effects. Points are coloured based on treatment, Disrupted (Betadine, Red), Control (Microbiome, Yellow), PC (Povidone, Blue) and the arrow indicate the direction and strength of significant selected predictors (Survival, shown in black).


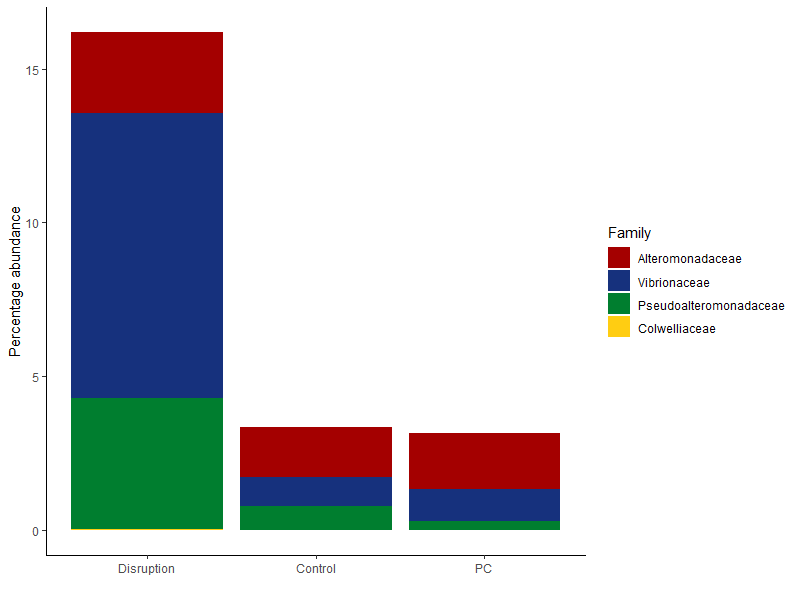


*Figure S2.* Percentage abundance of ASVs from the families Vibrionaceae, Alteromonadaceae, Pseudoalteromonadaceae and Colwelliaceae across treatments (Disruption with betadine, procedural control with povidone and control).


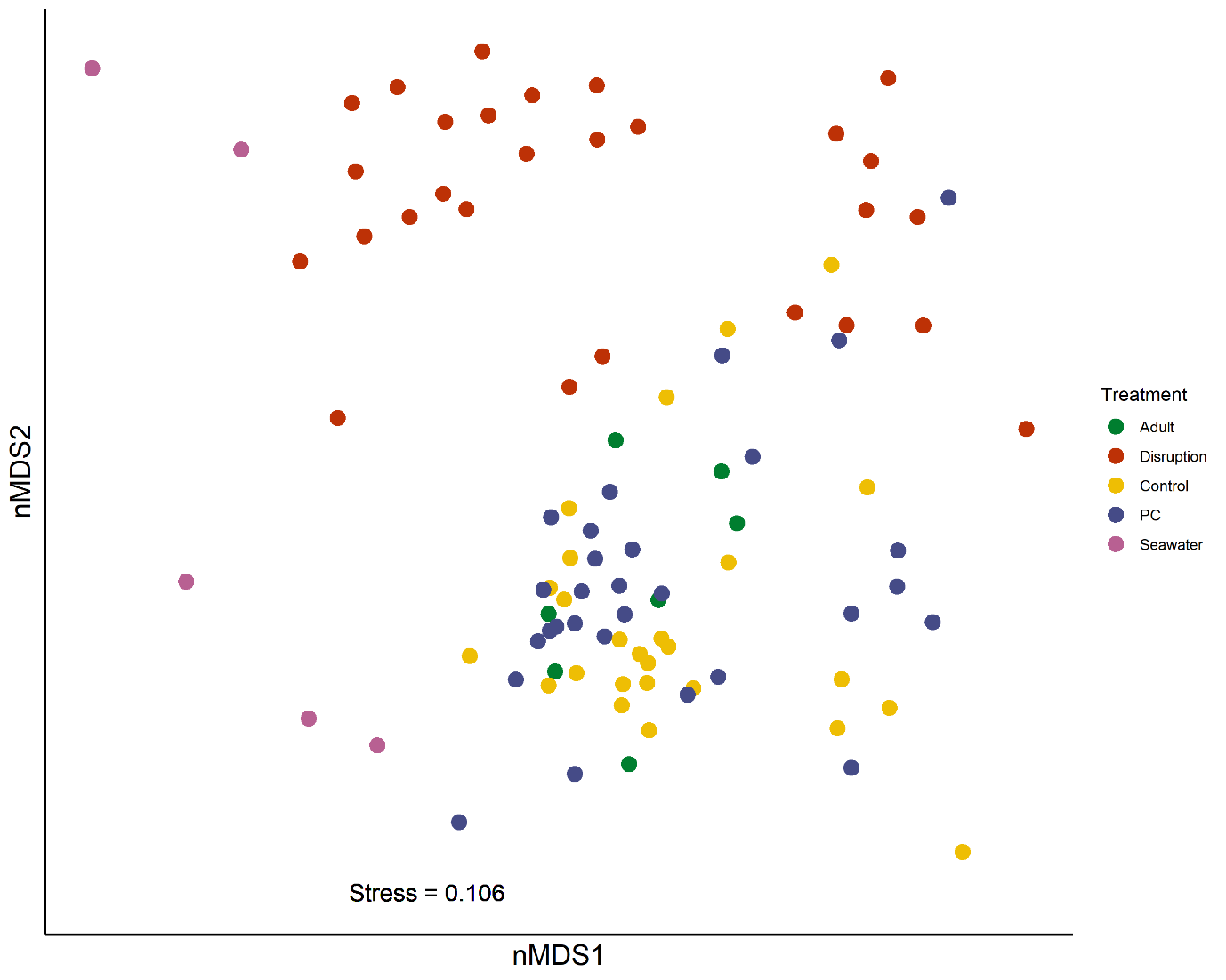


*Figure S3. Ecklonia radiata* gametophyte, adult and seawater microbiota community (Bray-Curtis on qPCR normalised, sqrt-transformed ASV abundances). Each dot represents the microbiota of gametophytes from each treatment (*n* = 30 - disruption (Red), control (Yellow), procedural control (Navy Blue), and *n* = 7 for adults (Green) and *n* = 5 and seawater (Purple)).


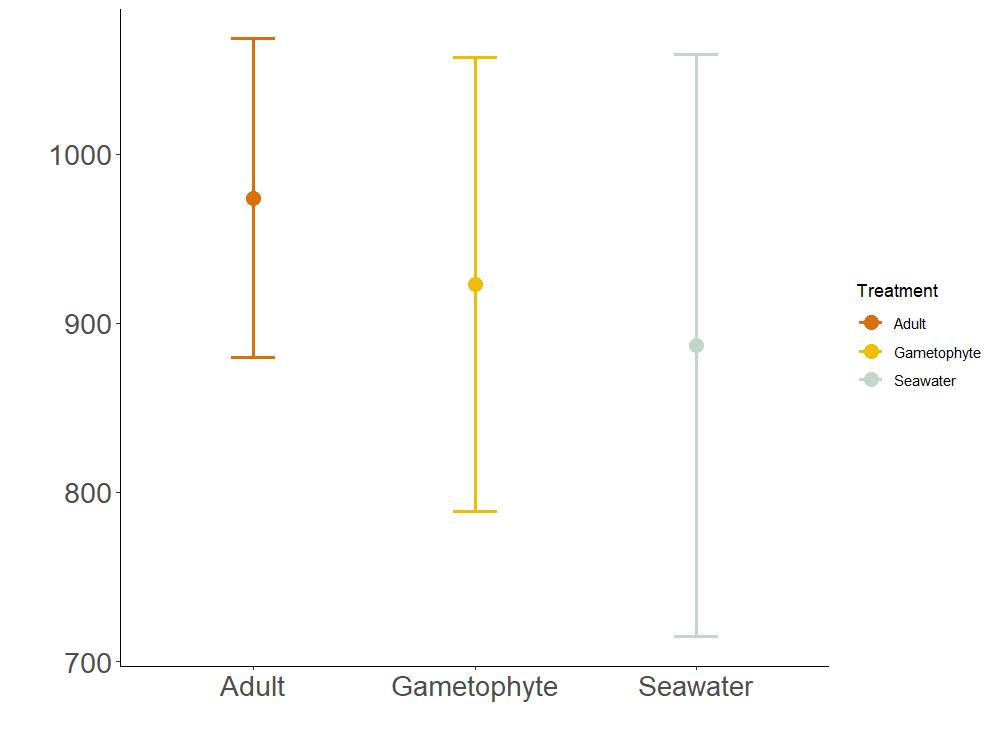


*Figure S4.* Observed richness among replicates within Adult, Seawater and Gametophytes. Dots represent the mean and bars represent standard error (*n* = 5). The gametophyte data for this plot was taken only from the undisturbed (control) treatment.


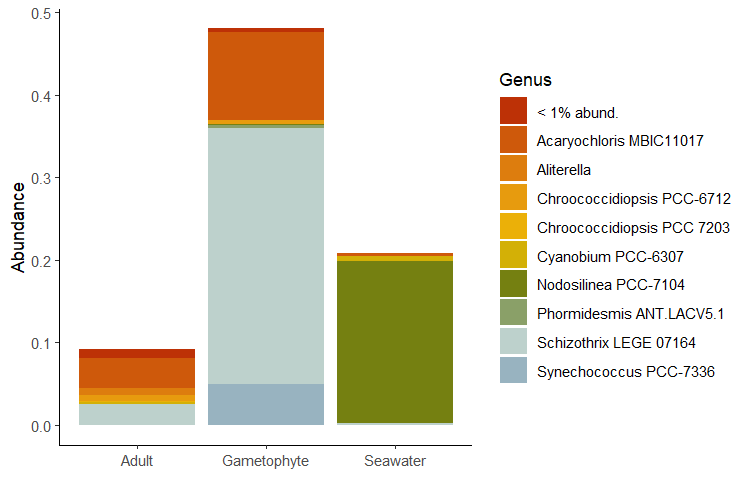
*Figure S5.*  Abundance of cyanobacteria genera for *Ecklonia* adult sporophytes, gametophytes, <1% abundance indicates cyanobacteria genera which were less than 1% abundance in each sample type.


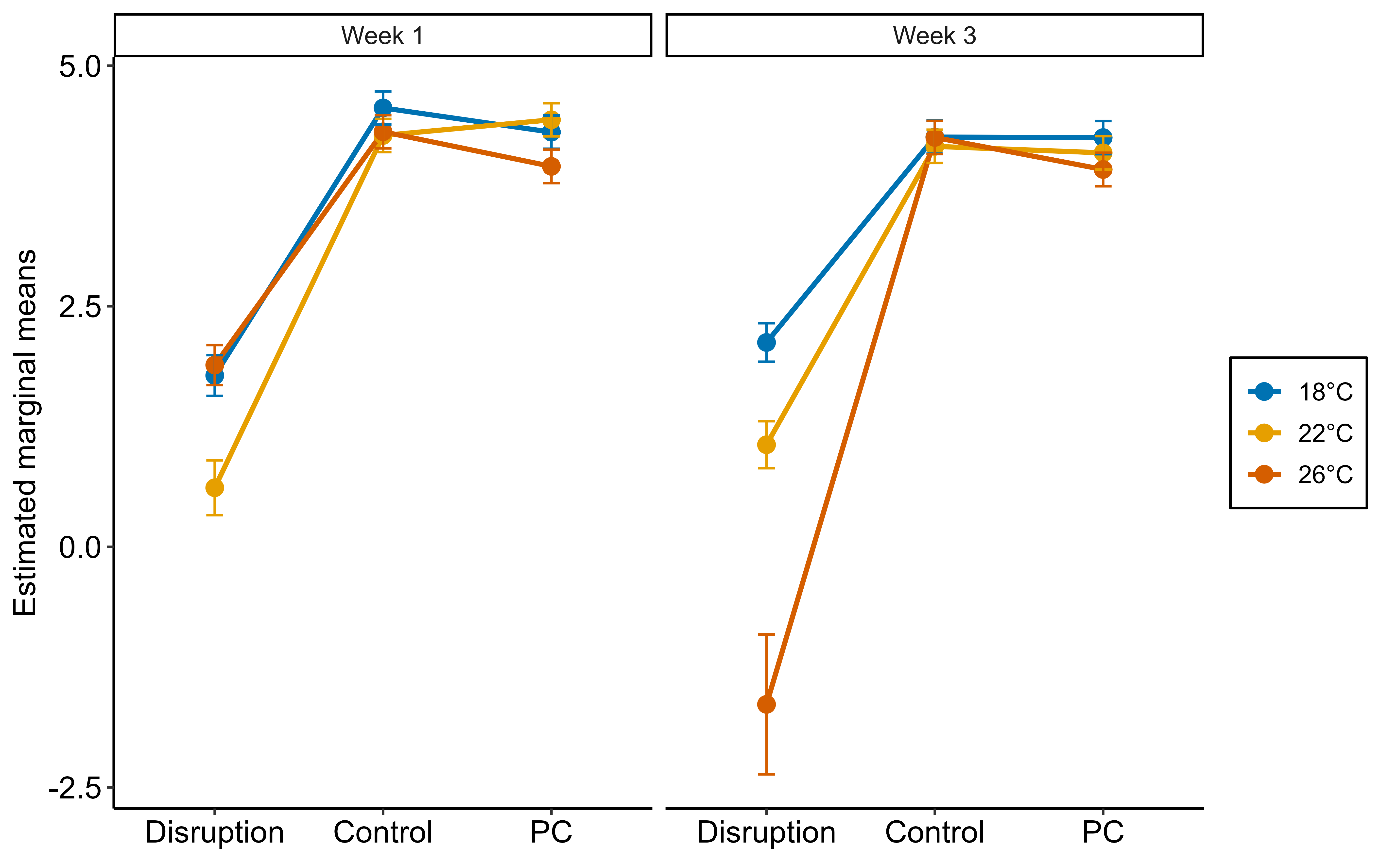


*Figure S6.* Interaction plot of the GLM relating survival of gametophytes to treatment (Disruption, control and procedural control), temperature (18°C, 22°C, 26°C) and timepoint (Week 1 and Week 3). The *y*-axis displays the normalized estimated marginal means calculated from the model, and error bars represent standard error of the marginal means. This is a visual display of the contrasts calculated to unravel the significant three-way interaction between treatment, temperature and timepoint specified by the model.


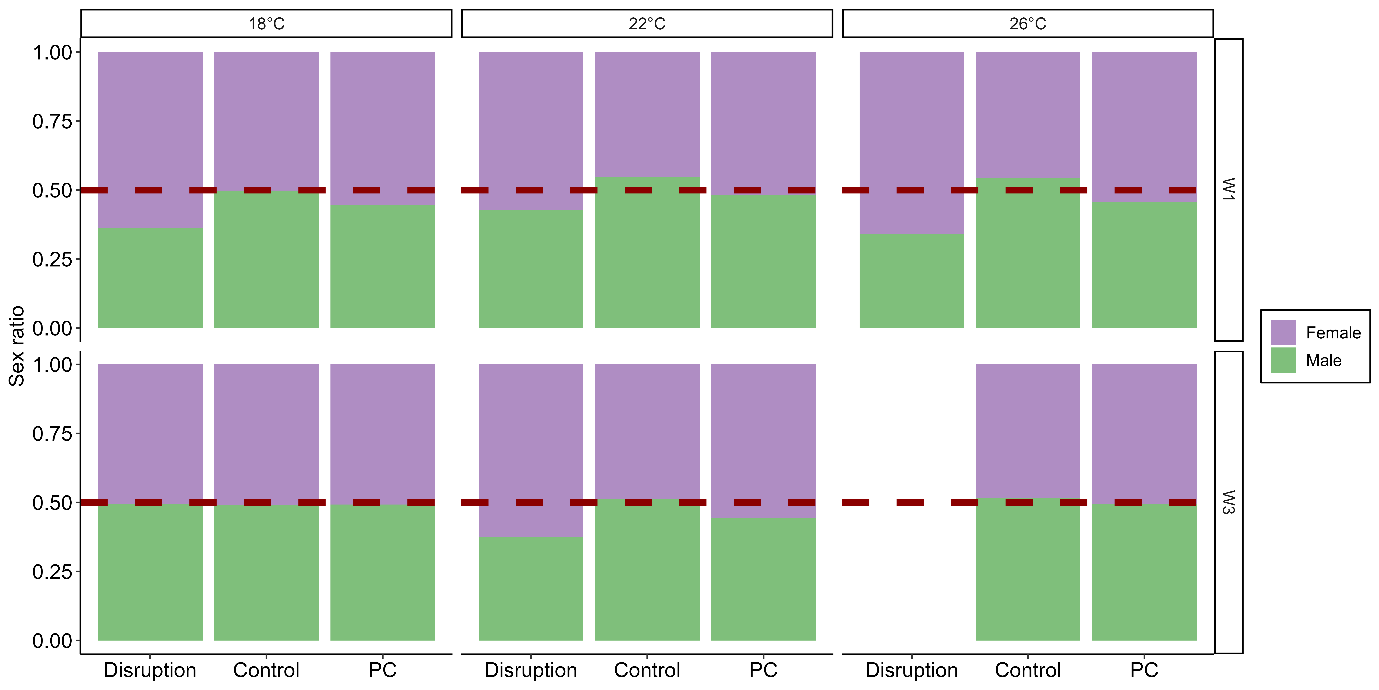


*Figure S7*. Sex ratios (total number of males and females) of *Ecklonia radiata* gametophytes under separate microbial treatments (Disruption with betadine, procedural control (PC) with povidone and control) and temperatures (18°C, 22°C, and 26°C) and timestamps (Week 1 and Week 3). The read dashed line indicates an equal number of males and females. Note no data is visible in the disruption treatment at 26°C, as no recognizable male or female gametophytes were identified.


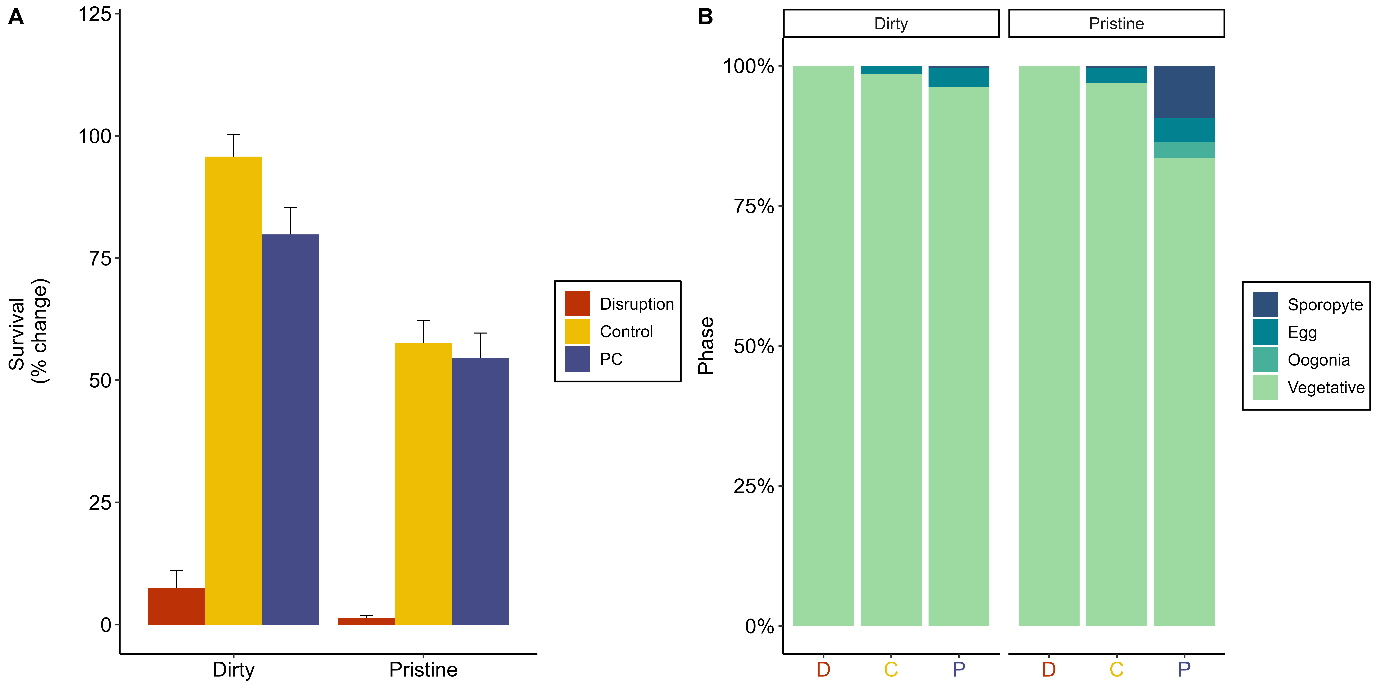


*Figure S8*. A) Mean survival rates (calculated as % change in abundance from initial settled spores to gametophytes) of *Ecklonia radiata* gametophytes under separate microbiota treatments (Disruption with betadine, procedural control with povidone and control) at sourced at two different collection sites. B) Phase of *Ecklonia radiata* female gametophyte development after three weeks as a percentage of total individuals for each treatment group (D = Disruption with betadine, P = procedural control with povidone and C = control) sourced at two different collection sites. ‘Dirty’ is inside a harbour structure, ‘Pristine’ outside. Sites were not replicated. Error bars represent standard error (*n* = 30).


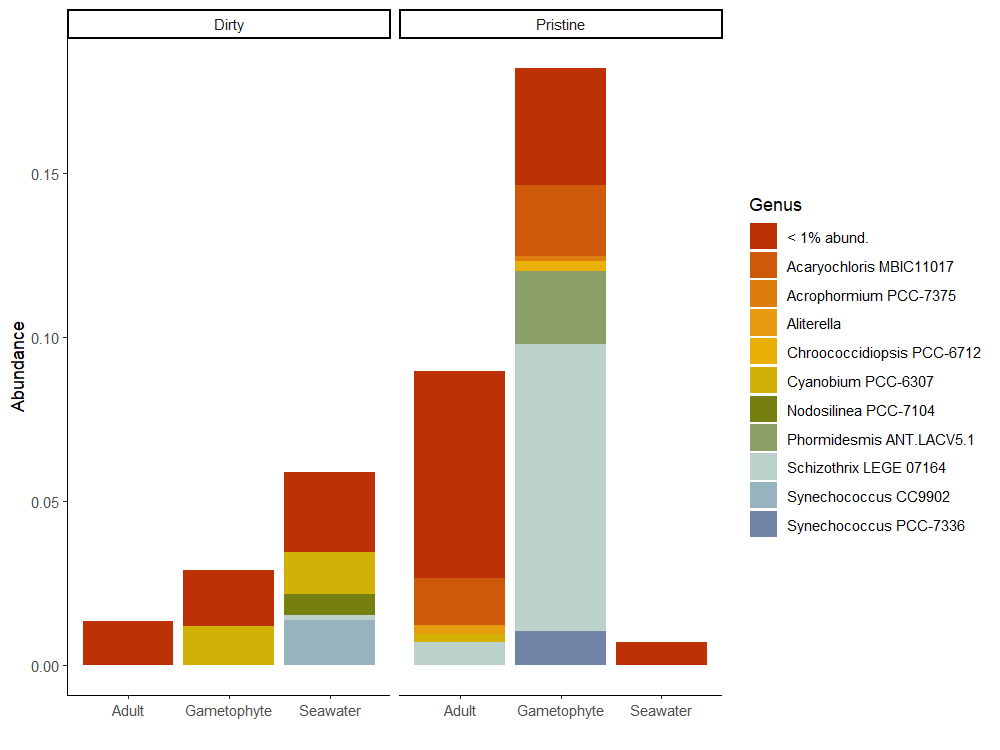


*Figure S9.* Abundance of Cyanobacteria genera for *Ecklonia* adult sporophytes, gametophytes and the seawater at two separate sites. ‘Dirty’ is inside a harbour structure, ‘Pristine’ outside. Sites were not replicated.

**Supplementary tables**

*Table S1*. Permutational analysis of variance based on Bray–Curtis similarity measures for qPCR-normalised, square-root transformed bacterial ASV abundances on *Ecklonia radiata* gametophytes among different disruption treatments (fixed, 3 levels: Disruption, Control and PC; *n* = 10; alpha = 0.05) and temperatures (fixed, crossed: 18, 22, 26^o^C). Post-hoc contrasts were calculated using the pairwise.adonis R function and p-values adjusted for multiple-testing.

|  |  | | | | | |
| --- | --- | --- | --- | --- | --- | --- |
| **Model** |  | *Df* | *SS* | *F* | *P* |  |
|  | Temperature | 2 | 3.4284 | 5.8267 | **0.001** |  |
|  | Treatment | 2 | 0.6664 | 1.1325 | **0.001** |  |
|  | Temperature X Treatment | 4 | 0.8039 | 0.6831 | 0.036 |  |
|  | Residual | 74 | 21.7707 |  |  |  |
|  | Total | 82 | 26.6694 |  |  |  |
| **Pairwise comparisons – Treatment X Temperature** | **Disrupted 18 = Disrupted 22 ≠ Disrupted 26 = Control 26 = PC 26 ≠ Control 22 = PC 22 = Control 18 = PC 18** | | | | |  |

*Table S2.* dbRDA Analyses for qPCR-normalised, square-root transformed bacterial ASV abundances on *Ecklonia radiata* adults and gametophytes among different host factors (fixed, 5 levels: Survival, Fertility, Sex ratio, Length and Abundance; *n* = 30, alpha = 0.05).

|  | *Inertia* | *Proportion* | *Rank* | *RealDims* |
| --- | --- | --- | --- | --- |
| Total | 26.6694 | 1 |  |  |
| Constrained | 18.83 | 0.7203 | 10 | 10 |
| Unconstrained | 7.839 | 0.1832 | 72 | 51 |
|  |  |  |  |  |
|  | *Df* | *SS* | *F* | *p* |
| Survival | 1 | 0.2666 | 0.7771 | **0.001** |
| Sex | 1 | 0.2013 | 1.2987 | 0.610 |
| Fertility | 1 | 0.4367 | 1.0298 | 0.436 |
| Length | 1 | 0.0982 | 1.7432 | 0.949 |
| Abundance | 1 | 0.9030 | 1.6861 | 0.109 |
|  |  |  |  |  |
| Residual | 72 | 20.1304 |  |  |
|  |  |  |  |  |

*Table S3*. Analyses of Sympsons diversity index and Richness of the bacterial community (qPCR-normalised, square-root transformed bacterial ASV abundances) on *Ecklonia radiata* gametophytes among different disruption treatments (fixed, 3 levels: Disruption, Control and PC; *n* = 10, alpha = 0.05) and temperatures (fixed, crossed: 18, 22, 26^o^C) and site (random: Clean, Dirty)

|  |  | *Df* | *SS* | *F* | *p* |
| --- | --- | --- | --- | --- | --- |
| Simpsons | Temperature | 3 | 2.230 | 1.670 | **0.043** |
|  | Treatment | 5 | 4.144 | 9.918 | **0.001** |
|  | Temperature X Treatment | 4 | 1.755 | 11.667 | **0.002** |
|  | Residual | 86 | 16.347 |  |  |
| Pairwise comparisons Temperature X Treatment | **Temperature 18 – Control = PC > Disruption**  **Temperature 22 – Control = PC > Disruption**  **Temperature 26 – Control = PC = Disruption** | | | | |
| Richness | Temperature | 3 | 5.238 | 18.735 | **0.043** |
|  | Treatment | 5 | 0.9876 | 3.5322 | **0.001** |
|  | Temperature X Treatment | 4 | 0.225 | 1.235 | **0.002** |
|  | Residual | 86 | 19.231 |  |  |
| Pairwise comparisons Temperature X Treatment | **Disrupted 18 = Disrupted 22 = Disrupted 26 < Control 26 = PC 26 < Control 22 = PC 22 = Control 18 = PC 18** | | | | |

*Table S5.* Permutational analysis of variance based on Bray–Curtis similarity measures for qPCR-normalised, square-root transformed bacterial ASV abundances on *Ecklonia radiata* adults and gametophytes among different types of swabbed environment (fixed, 3 levels: Adult, Gametophyte and Seawater ; *n* = 5, alpha = 0.05). Post-hoc contrasts were calculated using the pairwise.adonis R function and p-values adjusted for multiple-testing.

|  | *Df* | *SS* | *F* | *p* |
| --- | --- | --- | --- | --- |
| Type | 2 | 1.005 | 1.644 | **0.048** |
| Residual | 14 | 10.388 |  |  |
| Total | 16 | 11.393 |  |  |
| Pairwise Comparison | Adult = Gametophyte ≠ Seawater | | | |

*Table S4***.** Multivariate generalised linear model results showing significant ASVs associated with *Ecklonia radiata* gametophytes between treatments (fixed, 3 levels: Disruption, Control and PC; *n* = 10) (alpha = 0.05) and temperatures (Fixed, crossed: 18, 22, 26).

|  | | | | | | | |  |  |
| --- | --- | --- | --- | --- | --- | --- | --- | --- | --- |
| **ASV** | **Temp** | **Swab** | **Interaction** | **Kingdom** | **Phylum** | **Class** | **Order** | **Family** | **Genus** |
| ASV7554 | 0.001 | 0.368 | 0.001 | Bacteria | Proteobacteria | Alphaproteobacteria | Rhizobiales | Stappiaceae | Polymorphum |
| ASV7555 | 0.001 | 0.368 | 0.001 | Bacteria | Proteobacteria | Alphaproteobacteria | Rhizobiales | Stappiaceae | Polymorphum |
| ASV9109 | 0.001 | 0.368 | 0.001 | Bacteria | Proteobacteria | Gammaproteobacteria | Enterobacterales | Alteromonadaceae | Alteromonas |
| ASV9886 | 0.001 | 0.368 | 0.001 | Bacteria | Proteobacteria | Gammaproteobacteria | Enterobacterales | Alteromonadaceae | Alteromonas |
| ASV9885 | 0.001 | 0.368 | 0.001 | Bacteria | Proteobacteria | Gammaproteobacteria | Enterobacterales | Vibrionaceae | Candidatus Photodesmus |
| ASV9111 | 0.001 | 0.368 | 0.001 | Bacteria | Proteobacteria | Gammaproteobacteria | Pseudomonadales | Halomonadaceae | Cobetia |
| ASV9884 | 0.001 | 0.368 | 0.001 | Bacteria | Proteobacteria | Gammaproteobacteria | Pseudomonadales | Halomonadaceae | Cobetia |
| ASV8177 | 0.001 | 0.368 | 0.001 | Bacteria | Proteobacteria | Gammaproteobacteria | Pseudomonadales | Halomonadaceae | Halomonas |
| ASV9112 | 0.001 | 0.368 | 0.001 | Bacteria | Proteobacteria | Gammaproteobacteria | Pseudomonadales | Halomonadaceae | Halomonas |
| ASV8176 | 0.001 | 0.368 | 0.001 | Bacteria | Proteobacteria | Gammaproteobacteria | Enterobacterales | Pseudoalteromonadaceae | Pseudoalteromonas |
| ASV9110 | 0.001 | 0.368 | 0.001 | Bacteria | Proteobacteria | Gammaproteobacteria | Enterobacterales | Pseudoalteromonadaceae | Pseudoalteromonas |
| ASV2 | 0.041 | 0.047 | 0.001 | Bacteria | Proteobacteria | Gammaproteobacteria | Enterobacterales | Pseudoalteromonadaceae | Pseudoalteromonas |
| ASV5 | 0.041 | 0.047 | 0.001 | Bacteria | Proteobacteria | Gammaproteobacteria | Enterobacterales | Vibrionaceae | Vibrio |
| ASV3 | 0.041 | 0.055 | 0.001 | Bacteria | Proteobacteria | Gammaproteobacteria | Pseudomonadales | Halomonadaceae | Halomonas |
| ASV8 | 0.041 | 0.056 | 0.001 | Bacteria | Proteobacteria | Gammaproteobacteria | Pseudomonadales | Halomonadaceae | Halomonas |
| ASV4 | 0.041 | 0.056 | 0.001 | Bacteria | Proteobacteria | Gammaproteobacteria | Pseudomonadales | Halomonadaceae | Halomonas |
| ASV12 | 0.111 | 0.001 | 0.001 | Bacteria | Proteobacteria | Alphaproteobacteria | Rhizobiales | Stappiaceae | Labrenzia |
| ASV23 | 0.111 | 0.001 | 0.001 | Bacteria | Proteobacteria | Alphaproteobacteria | Rhizobiales | Stappiaceae | Labrenzia |
| ASV98 | 0.111 | 0.008 | 0.001 | Bacteria | Proteobacteria | Gammaproteobacteria | Enterobacterales | Vibrionaceae | Aliivibrio |
| ASV172 | 0.132 | 0.126 | 0.001 | Bacteria | Cyanobacteria | Cyanobacteriia | Thermosynechococcales | Acaryochloridaceae | Acaryochloris MBIC11017 |
| ASV326 | 0.132 | 0.126 | 0.001 | Bacteria | Cyanobacteria | Cyanobacteriia | Thermosynechococcales | Acaryochloridaceae | Acaryochloris MBIC11017 |
| ASV20 | 0.136 | 0.001 | 0.001 | Bacteria | Proteobacteria | Gammaproteobacteria | Enterobacterales | Vibrionaceae | Vibrio |
| ASV4183 | 0.227 | 0.352 | 0.001 | Bacteria | Bacteroidota | Bacteroidia | Flavobacteriales | Flavobacteriaceae | Actibacter |
| ASV2034 | 0.308 | 0.345 | 0.001 | Bacteria | Cyanobacteria | Cyanobacteriia | Cyanobacteriales | Microcystaceae | Synechocystis BDHKU-20401 |
| ASV2020 | 0.323 | 0.35 | 0.001 | Bacteria | Cyanobacteria | Cyanobacteriia | Cyanobacteriales | Xenococcaceae | Pleurocapsa PCC-7319 |
| ASV1552 | 0.342 | 0.349 | 0.001 | Bacteria | Cyanobacteria | Cyanobacteriia | Cyanobacteriales | Nostocaceae | Calothrix PCC-6303 |
| ASV2191 | 0.347 | 0.092 | 0.036 | Bacteria | Cyanobacteria | Cyanobacteriia | Synechococcales | Cyanobiaceae | Cyanobium PCC-6307 |
| ASV539 | 0.356 | 0.351 | 0.001 | Bacteria | Cyanobacteria | Cyanobacteriia | Cyanobacteriales | Xenococcaceae | Pleurocapsa PCC-7319 |
| ASV656 | 0.356 | 0.351 | 0.001 | Bacteria | Cyanobacteria | Cyanobacteriia | Cyanobacteriales | Xenococcaceae | Pleurocapsa PCC-7319 |
| ASV26 | 0.357 | 0.003 | 0.001 | Bacteria | Proteobacteria | Gammaproteobacteria | Pseudomonadales | Marinomonadaceae | Marinomonas |
| ASV587 | 0.358 | 0.352 | 0.001 | Bacteria | Cyanobacteria | Cyanobacteriia | Cyanobacteriales | Chroococcidiopsaceae | Aliterella |
| ASV771 | 0.36 | 0.35 | 0.001 | Bacteria | Cyanobacteria | Cyanobacteriia | Synechococcales | Cyanobiaceae | Cyanobium PCC-6307 |
| ASV230 | 0.362 | 0.352 | 0.001 | Bacteria | Cyanobacteria | Cyanobacteriia | Cyanobacteriales | Xenococcaceae | Pleurocapsa PCC-7319 |
| ASV258 | 0.362 | 0.351 | 0.001 | Bacteria | Cyanobacteria | Cyanobacteriia | Cyanobacteriales | Xenococcaceae | Pleurocapsa PCC-7319 |
| ASV84 | 0.366 | 0.001 | 0.001 | Bacteria | Proteobacteria | Gammaproteobacteria | Enterobacterales | Vibrionaceae | Vibrio |
| ASV82 | 0.367 | 0.355 | 0.001 | Bacteria | Proteobacteria | Gammaproteobacteria | Enterobacterales | Pseudoalteromonadaceae | Pseudoalteromonas |
| ASV6971 | 0.367 | 0.368 | 0.001 | Bacteria | Proteobacteria | Gammaproteobacteria | Enterobacterales | Pseudoalteromonadaceae | Pseudoalteromonas |
| ASV100 | 0.367 | 0.001 | 0.001 | Bacteria | Proteobacteria | Gammaproteobacteria | Enterobacterales | Vibrionaceae | Vibrio |
| ASV58 | 0.367 | 0.001 | 0.001 | Bacteria | Bacteroidota | Bacteroidia | Flavobacteriales | Flavobacteriaceae | Dokdonia |
| ASV100 | 0.367 | 0.001 | 0.001 | Bacteria | Proteobacteria | Gammaproteobacteria | Enterobacterales | Vibrionaceae | Vibrio |
| ASV301 | 0.367 | 0.001 | 0.001 | Bacteria | Proteobacteria | Gammaproteobacteria | Enterobacterales | Vibrionaceae | Vibrio |
| ASV116 | 0.367 | 0.004 | 0.001 | Bacteria | Proteobacteria | Gammaproteobacteria | Enterobacterales | Alteromonadaceae | Alteromonas |
| ASV2855 | 0.368 | 0.063 | 0.036 | Bacteria | Proteobacteria | Gammaproteobacteria | Enterobacterales | Vibrionaceae | Aliivibrio |
| ASV454 | 0.378 | 0.992 | 0.001 | Bacteria | Cyanobacteria | Cyanobacteriia | Phormidesmiales | Nodosilineaceae | Nodosilinea PCC-7104 |
| ASV926 | 0.386 | 0.938 | 0.001 | Bacteria | Cyanobacteria | Cyanobacteriia | Eurycoccales | Eurycoccales Incertae Sedis | Synechococcus PCC-7336 |
| ASV2597 | 0.415 | 0.018 | 0.036 | Bacteria | Proteobacteria | Gammaproteobacteria | Enterobacterales | Vibrionaceae | Photobacterium |
| ASV2597 | 0.415 | 0.018 | 0.036 | Bacteria | Proteobacteria | Gammaproteobacteria | Enterobacterales | Vibrionaceae | Photobacterium |
| ASV97 | 0.775 | 0.046 | 0.001 | Bacteria | Proteobacteria | Gammaproteobacteria | Enterobacterales | Vibrionaceae | Vibrio |
| ASV110 | 0.873 | 0.579 | 0.001 | Bacteria | Proteobacteria | Gammaproteobacteria | Enterobacterales | Vibrionaceae | Vibrio |
| ASV100 | 1 | 0.001 | 0.919 | Bacteria | Proteobacteria | Gammaproteobacteria | Enterobacterales | Vibrionaceae | Vibrio |
| ASV12 | 1 | 0.001 | 1 | Bacteria | Proteobacteria | Alphaproteobacteria | Rhizobiales | Stappiaceae | Labrenzia |
| ASV130 | 1 | 0.001 | 1 | Bacteria | Proteobacteria | Gammaproteobacteria | Enterobacterales | Vibrionaceae | Vibrio |
| ASV217 | 1 | 0.001 | 1 | Bacteria | Proteobacteria | Alphaproteobacteria | Rhizobiales | Stappiaceae | Labrenzia |
| ASV301 | 1 | 0.001 | 0.99 | Bacteria | Proteobacteria | Gammaproteobacteria | Enterobacterales | Vibrionaceae | Vibrio |
| ASV54 | 1 | 0.001 | 0.997 | Bacteria | Proteobacteria | Gammaproteobacteria | Enterobacterales | Pseudoalteromonadaceae | Pseudoalteromonas |
| ASV57 | 1 | 0.001 | 1 | Bacteria | Proteobacteria | Alphaproteobacteria | Rhodobacterales | Rhodobacteraceae | NA |
| ASV58 | 1 | 0.001 | 1 | Bacteria | Bacteroidota | Bacteroidia | Flavobacteriales | Flavobacteriaceae | Dokdonia |
| ASV84 | 1 | 0.001 | 0.98 | Bacteria | Proteobacteria | Gammaproteobacteria | Enterobacterales | Vibrionaceae | Vibrio |
| ASV906 | 1 | 0.001 | 1 | Bacteria | Proteobacteria | Gammaproteobacteria | Enterobacterales | Vibrionaceae | Aliivibrio |
| ASV6 | 5 | 0.001 | 0.125 | Bacteria | Proteobacteria | Gammaproteobacteria | Enterobacterales | Vibrionaceae | Vibrio |
| ASV9 | 8 | 0.001 | 0.144 | Bacteria | Proteobacteria | Alphaproteobacteria | Rhizobiales | Stappiaceae | Labrenzia |
| ASV12 | 11 | 0.001 | 0.001 | Bacteria | Proteobacteria | Alphaproteobacteria | Rhizobiales | Stappiaceae | Labrenzia |
| ASV20 | 14 | 0.001 | 0.001 | Bacteria | Proteobacteria | Gammaproteobacteria | Enterobacterales | Vibrionaceae | Vibrio |
| ASV23 | 17 | 0.001 | 0.001 | Bacteria | Proteobacteria | Alphaproteobacteria | Rhizobiales | Stappiaceae | Labrenzia |
| ASV27 | 20 | 0.001 | 0.364 | Bacteria | Proteobacteria | Alphaproteobacteria | Rhizobiales | Rhizobiaceae | Lentilitoribacter |
| ASV35 | 23 | 0.001 | 0.137 | Bacteria | Proteobacteria | Alphaproteobacteria | Kiloniellales | Kiloniellaceae | Pelagibius |
| ASV43 | 27 | 0.001 | 0.373 | Bacteria | Bacteroidota | Bacteroidia | Flavobacteriales | Flavobacteriaceae | Croceitalea |
| ASV44 | 28 | 0.001 | 0.137 | Bacteria | Proteobacteria | Alphaproteobacteria | Rhodobacterales | Rhodobacteraceae | Thalassobius |
| ASV45 | 29 | 0.001 | 0.369 | Bacteria | Proteobacteria | Gammaproteobacteria | Pseudomonadales | Alcanivoracaceae1 | Alcanivorax |
| ASV51 | 32 | 0.001 | 0.126 | Bacteria | Bacteroidota | Bacteroidia | Flavobacteriales | Flavobacteriaceae | Aquimarina |
| ASV56 | 34 | 0.001 | 0.134 | Bacteria | Proteobacteria | Alphaproteobacteria | Sphingomonadales | Sphingomonadaceae | Sphingorhabdus |
| ASV59 | 37 | 0.001 | 0.125 | Bacteria | Proteobacteria | Alphaproteobacteria | Rhodobacterales | Rhodobacteraceae | Ruegeria |
| ASV71 | 44 | 0.001 | 0.364 | Bacteria | Proteobacteria | Gammaproteobacteria | Pseudomonadales | Halomonadaceae | Halomonas |
| ASV72 | 45 | 0.001 | 0.367 | Bacteria | Proteobacteria | Gammaproteobacteria | Pseudomonadales | Halomonadaceae | Halomonas |
| ASV73 | 46 | 0.001 | 0.364 | Bacteria | Proteobacteria | Gammaproteobacteria | Pseudomonadales | Halomonadaceae | Halomonas |
| ASV77 | 49 | 0.001 | 0.365 | Bacteria | Proteobacteria | Gammaproteobacteria | Pseudomonadales | Halomonadaceae | Halomonas |
| ASV79 | 51 | 0.001 | 0.373 | Bacteria | Proteobacteria | Alphaproteobacteria | Sphingomonadales | Sphingomonadaceae | Sphingopyxis |
| ASV81 | 53 | 0.001 | 0.373 | Bacteria | Proteobacteria | Alphaproteobacteria | Caulobacterales | Hyphomonadaceae | Maricaulis |
| ASV85 | 57 | 0.001 | 0.142 | Bacteria | Proteobacteria | Gammaproteobacteria | Pseudomonadales | Halomonadaceae | Halomonas |
| ASV86 | 58 | 0.001 | 0.345 | Bacteria | Proteobacteria | Alphaproteobacteria | Rhodobacterales | Rhodobacteraceae | Octadecabacter |
| ASV87 | 59 | 0.001 | 0.142 | Bacteria | Proteobacteria | Gammaproteobacteria | Pseudomonadales | Halomonadaceae | Halomonas |
| ASV96 | 64 | 0.001 | 0.717 | Bacteria | Proteobacteria | Gammaproteobacteria | Pseudomonadales | Halomonadaceae | Halomonas |
| ASV98 | 66 | 0.001 | 0.008 | Bacteria | Proteobacteria | Gammaproteobacteria | Enterobacterales | Vibrionaceae | Aliivibrio |
| ASV102 | 69 | 0.001 | 0.731 | Bacteria | Proteobacteria | Gammaproteobacteria | Pseudomonadales | Halomonadaceae | Halomonas |
| ASV129 | 77 | 0.001 | 0.366 | Bacteria | Bacteroidota | Bacteroidia | Flavobacteriales | Flavobacteriaceae | Flagellimonas |
| ASV139 | 85 | 0.001 | 0.376 | Bacteria | Proteobacteria | Alphaproteobacteria | Parvibaculales | Parvibaculaceae | Parvibaculum |
| ASV172 | 103 | 0.001 | 0.126 | Bacteria | Cyanobacteria | Cyanobacteriia | Thermosynechococcales | Acaryochloridaceae | Acaryochloris MBIC11017 |
| ASV173 | 104 | 0.001 | 0.992 | Bacteria | Proteobacteria | Gammaproteobacteria | Enterobacterales | Vibrionaceae | Aliivibrio |
| ASV189 | 114 | 0.001 | 0.368 | Bacteria | Proteobacteria | Alphaproteobacteria | Rhodobacterales | Rhodobacteraceae | Octadecabacter |
| ASV231 | 126 | 0.001 | 0.126 | Bacteria | Proteobacteria | Alphaproteobacteria | Rhodobacterales | Rhodobacteraceae | Jannaschia |
| ASV280 | 149 | 0.001 | 0.368 | Bacteria | Bacteroidota | Bacteroidia | Flavobacteriales | Flavobacteriaceae | Algibacter |
| ASV292 | 152 | 0.001 | 0.124 | Bacteria | Proteobacteria | Gammaproteobacteria | Arenicellales | Arenicellaceae | Arenicella |
| ASV326 | 170 | 0.001 | 0.126 | Bacteria | Cyanobacteria | Cyanobacteriia | Thermosynechococcales | Acaryochloridaceae | Acaryochloris MBIC11017 |
| ASV337 | 174 | 0.001 | 0.351 | Bacteria | Proteobacteria | Gammaproteobacteria | Granulosicoccales | Granulosicoccaceae | Granulosicoccus |
| ASV494 | 223 | 0.001 | 0.352 | Bacteria | Proteobacteria | Alphaproteobacteria | Caulobacterales | Hyphomonadaceae | Hellea |
| ASV562 | 243 | 0.001 | 0.35 | Bacteria | Proteobacteria | Gammaproteobacteria | Thiotrichales | Thiotrichaceae | Cocleimonas |
| ASV574 | 247 | 0.001 | 0.352 | Bacteria | Verrucomicrobiota | Verrucomicrobiae | Verrucomicrobiales | Rubritaleaceae | Rubritalea |
| ASV627 | 256 | 0.001 | 0.351 | Bacteria | Proteobacteria | Gammaproteobacteria | Thiotrichales | Thiotrichaceae | Leucothrix |
| ASV683 | 271 | 0.001 | 0.376 | Bacteria | Proteobacteria | Alphaproteobacteria | Sphingomonadales | Sphingomonadaceae | Erythrobacter |
| ASV756 | 292 | 0.001 | 0.351 | Bacteria | Bacteroidota | Bacteroidia | Flavobacteriales | Flavobacteriaceae | Algitalea |
| ASV758 | 294 | 0.001 | 0.352 | Bacteria | Bacteroidota | Bacteroidia | Chitinophagales | Saprospiraceae | Portibacter |
| ASV774 | 297 | 0.001 | 0.352 | Bacteria | Proteobacteria | Gammaproteobacteria | Granulosicoccales | Granulosicoccaceae | Granulosicoccus |
| ASV781 | 302 | 0.001 | 0.35 | Bacteria | Verrucomicrobiota | Verrucomicrobiae | Verrucomicrobiales | Rubritaleaceae | Roseibacillus |
| ASV823 | 314 | 0.001 | 0.352 | Bacteria | Proteobacteria | Gammaproteobacteria | Arenicellales | Arenicellaceae | Perspicuibacter |
| ASV861 | 325 | 0.001 | 0.352 | Bacteria | Bacteroidota | Bacteroidia | Flavobacteriales | Flavobacteriaceae | Algitalea |
| ASV973 | 343 | 0.001 | 0.351 | Bacteria | Proteobacteria | Gammaproteobacteria | Arenicellales | Arenicellaceae | Arenicella |
| ASV999 | 352 | 0.001 | 0.352 | Bacteria | Verrucomicrobiota | Verrucomicrobiae | Verrucomicrobiales | Rubritaleaceae | Rubritalea |
| ASV1028 | 360 | 0.001 | 0.351 | Bacteria | Proteobacteria | Gammaproteobacteria | Pseudomonadales | Thioglobaceae | IheB2-31 |
| ASV1043 | 363 | 0.001 | 0.999 | Bacteria | Proteobacteria | Gammaproteobacteria | Thiotrichales | Thiotrichaceae | Cocleimonas |
| ASV1137 | 372 | 0.001 | 0.351 | Bacteria | Bacteroidota | Bacteroidia | Flavobacteriales | Flavobacteriaceae | Winogradskyella |
| ASV1141 | 373 | 0.001 | 0.347 | Bacteria | Proteobacteria | Gammaproteobacteria | Thiotrichales | Thiotrichaceae | Thiothrix |
| ASV1195 | 380 | 0.001 | 0.372 | Bacteria | Bacteroidota | Bacteroidia | Flavobacteriales | Cryomorphaceae | Owenweeksia |
| ASV1213 | 383 | 0.001 | 0.352 | Bacteria | Bacteroidota | Bacteroidia | Flavobacteriales | Flavobacteriaceae | Aurantivirga |
| ASV1216 | 384 | 0.001 | 0.352 | Bacteria | Proteobacteria | Gammaproteobacteria | Arenicellales | Arenicellaceae | Arenicella |
| ASV1240 | 391 | 0.001 | 0.347 | Bacteria | Bacteroidota | Bacteroidia | Flavobacteriales | Flavobacteriaceae | Tenacibaculum |
| ASV1255 | 392 | 0.001 | 0.351 | Bacteria | Bacteroidota | Bacteroidia | Chitinophagales | Saprospiraceae | Aureispira |
| ASV1284 | 399 | 0.001 | 0.352 | Bacteria | Proteobacteria | Gammaproteobacteria | Granulosicoccales | Granulosicoccaceae | Granulosicoccus |
| ASV1320 | 403 | 0.001 | 0.376 | Bacteria | Bacteroidota | Bacteroidia | Flavobacteriales | Flavobacteriaceae | Winogradskyella |
| ASV1325 | 404 | 0.001 | 0.351 | Bacteria | Planctomycetota | Planctomycetes | Planctomycetales | Rubinisphaeraceae | Fuerstia |
| ASV1334 | 406 | 0.001 | 0.352 | Bacteria | Proteobacteria | Gammaproteobacteria | Granulosicoccales | Granulosicoccaceae | Granulosicoccus |
| ASV1409 | 412 | 0.001 | 0.349 | Bacteria | Bacteroidota | Bacteroidia | Flavobacteriales | Flavobacteriaceae | Polaribacter |
| ASV1449 | 417 | 0.001 | 0.352 | Bacteria | Bacteroidota | Bacteroidia | Flavobacteriales | Flavobacteriaceae | Algitalea |
| ASV1469 | 418 | 0.001 | 0.352 | Bacteria | Proteobacteria | Gammaproteobacteria | Arenicellales | Arenicellaceae | Arenicella |
| ASV1497 | 421 | 0.001 | 0.352 | Bacteria | Bacteroidota | Bacteroidia | Flavobacteriales | Flavobacteriaceae | Tenacibaculum |
| ASV1498 | 422 | 0.001 | 0.352 | Bacteria | Bacteroidota | Bacteroidia | Flavobacteriales | Flavobacteriaceae | Tenacibaculum |
| ASV1540 | 428 | 0.001 | 0.352 | Bacteria | Proteobacteria | Gammaproteobacteria | Arenicellales | Arenicellaceae | HTCC5015 |
| ASV1552 | 431 | 0.001 | 0.349 | Bacteria | Cyanobacteria | Cyanobacteriia | Cyanobacteriales | Nostocaceae | Calothrix PCC-6303 |
| ASV1560 | 432 | 0.001 | 0.352 | Bacteria | Bacteroidota | Bacteroidia | Chitinophagales | Saprospiraceae | Portibacter |
| ASV1587 | 435 | 0.001 | 0.35 | Bacteria | Bacteroidota | Bacteroidia | Flavobacteriales | Crocinitomicaceae | Crocinitomix |
| ASV1614 | 437 | 0.001 | 0.351 | Bacteria | Actinobacteriota | Acidimicrobiia | Microtrichales | Microtrichaceae | Sva0996 marine group |
| ASV1633 | 442 | 0.001 | 0.352 | Bacteria | Bacteroidota | Bacteroidia | Chitinophagales | Saprospiraceae | Lewinella |
| ASV1658 | 446 | 0.001 | 0.352 | Bacteria | Proteobacteria | Gammaproteobacteria | Francisellales | Francisellaceae | Francisella |
| ASV1659 | 447 | 0.001 | 0.352 | Bacteria | Bacteroidota | Bacteroidia | Chitinophagales | Saprospiraceae | Lewinella |
| ASV1726 | 452 | 0.001 | 0.35 | Bacteria | Proteobacteria | Alphaproteobacteria | Rhodobacterales | Rhodobacteraceae | Sulfitobacter |
| ASV1758 | 454 | 0.001 | 0.351 | Bacteria | Bacteroidota | Bacteroidia | Flavobacteriales | Flavobacteriaceae | Algitalea |
| ASV1773 | 456 | 0.001 | 0.351 | Bacteria | Proteobacteria | Alphaproteobacteria | Caulobacterales | Hyphomonadaceae | Robiginitomaculum |
| ASV1776 | 458 | 0.001 | 0.352 | Bacteria | Proteobacteria | Gammaproteobacteria | Granulosicoccales | Granulosicoccaceae | Granulosicoccus |
| ASV1799 | 463 | 0.001 | 0.375 | Bacteria | Proteobacteria | Alphaproteobacteria | Sphingomonadales | Sphingomonadaceae | Altererythrobacter |
| ASV1806 | 464 | 0.001 | 0.352 | Bacteria | Proteobacteria | Gammaproteobacteria | Arenicellales | Arenicellaceae | Arenicella |
| ASV1807 | 465 | 0.001 | 0.349 | Bacteria | Proteobacteria | Gammaproteobacteria | Pseudomonadales | Cellvibrionaceae | Agaribacterium |
| ASV1856 | 472 | 0.001 | 0.352 | Bacteria | Bacteroidota | Bacteroidia | Flavobacteriales | Flavobacteriaceae | Aurantivirga |
| ASV2019 | 483 | 0.001 | 0.351 | Bacteria | Proteobacteria | Gammaproteobacteria | Pseudomonadales | Halieaceae | OM60(NOR5) clade |
| ASV2020 | 484 | 0.001 | 0.35 | Bacteria | Cyanobacteria | Cyanobacteriia | Cyanobacteriales | Xenococcaceae | Pleurocapsa PCC-7319 |
| ASV2034 | 486 | 0.001 | 0.345 | Bacteria | Cyanobacteria | Cyanobacteriia | Cyanobacteriales | Microcystaceae | Synechocystis BDHKU-20401 |
| ASV2195 | 499 | 0.001 | 0.352 | Bacteria | Bacteroidota | Bacteroidia | Flavobacteriales | Flavobacteriaceae | Winogradskyella |
| ASV2281 | 509 | 0.001 | 0.352 | Bacteria | Proteobacteria | Gammaproteobacteria | Granulosicoccales | Granulosicoccaceae | Granulosicoccus |
| ASV2282 | 510 | 0.001 | 0.376 | Bacteria | Bacteroidota | Bacteroidia | Flavobacteriales | Flavobacteriaceae | Maribacter |
| ASV2340 | 519 | 0.001 | 0.352 | Bacteria | Proteobacteria | Alphaproteobacteria | Rickettsiales | Rickettsiaceae | Candidatus Megaira |
| ASV2412 | 522 | 0.001 | 0.351 | Bacteria | Bacteroidota | Bacteroidia | Chitinophagales | Saprospiraceae | Lewinella |
| ASV2448 | 525 | 0.001 | 0.352 | Bacteria | Proteobacteria | Gammaproteobacteria | Thiotrichales | Thiotrichaceae | Leucothrix |
| ASV2449 | 526 | 0.001 | 0.352 | Bacteria | Bacteroidota | Bacteroidia | Flavobacteriales | Flavobacteriaceae | Wenyingzhuangia |
| ASV2545 | 532 | 0.001 | 0.352 | Bacteria | Proteobacteria | Alphaproteobacteria | Rhodobacterales | Rhodobacteraceae | Tateyamaria |
| ASV2801 | 554 | 0.001 | 0.351 | Bacteria | Deinococcota | Deinococci | Deinococcales | Trueperaceae | Truepera |
| ASV2988 | 562 | 0.001 | 0.352 | Bacteria | Proteobacteria | Gammaproteobacteria | Arenicellales | Arenicellaceae | HTCC5015 |
| ASV3151 | 567 | 0.001 | 0.352 | Bacteria | Proteobacteria | Alphaproteobacteria | Rhodobacterales | Rhodobacteraceae | Jannaschia |
| ASV3445 | 584 | 0.001 | 0.351 | Bacteria | Proteobacteria | Gammaproteobacteria | Thiotrichales | Thiotrichaceae | Leucothrix |
| ASV4183 | 619 | 0.001 | 0.352 | Bacteria | Bacteroidota | Bacteroidia | Flavobacteriales | Flavobacteriaceae | Actibacter |
| ASV4378 | 630 | 0.001 | 0.352 | Bacteria | Proteobacteria | Gammaproteobacteria | Arenicellales | Arenicellaceae | Perspicuibacter |
| ASV6234 | 718 | 0.001 | 0.352 | Bacteria | Bacteroidota | Bacteroidia | Flavobacteriales | Flavobacteriaceae | Dokdonia |
